# A single‐cell transcriptomic atlas characterizes age‐related changes of murine cranial stem cell niches

**DOI:** 10.1111/acel.13980

**Published:** 2023-09-08

**Authors:** Bo Li, Jingya Li, Bingzhi Li, Takehito Ouchi, Longjiang Li, Yu Li, Zhihe Zhao

**Affiliations:** ^1^ State Key Laboratory of Oral Diseases, National Center for Stomatology, National Clinical Research Center for Oral Diseases, Department of Orthodontics, West China Hospital of Stomatology Sichuan University Sichuan Chengdu China; ^2^ State Key Laboratory of Oral Diseases, National Center for Stomatology, National Clinical Research Center for Oral Diseases, Department of Head and Neck Oncology, West China Hospital of Stomatology Sichuan University Sichuan Chengdu China; ^3^ Department of Physiology Tokyo Dental College Tokyo Japan

**Keywords:** cranial bone marrow, inflammaging, mesenchymal stem cell, single‐cell RNA sequencing, stem cell niche, suture mesenchyme

## Abstract

The craniofacial bones provide structural support for the skull and accommodate the vulnerable brain tissue with a protective cavity. The bone tissue undergoes constant turnover, which relies on skeletal stem cells (SSCs) and/or mesenchymal stem cells (MSCs) and their niches. SSCs/MSCs and their perivascular niche within the bone marrow are well characterized in long bones. As for cranial bones, besides bone marrow, the suture mesenchyme has been identified as a unique niche for SSCs/MSCs of craniofacial bones. However, a comprehensive study of the two different cranial stem cell niches at single‐cell resolution is still lacking. In addition, during the progression of aging, age‐associated changes in cranial stem cell niches and resident cells remain uncovered. In this study, we investigated age‐related changes in cranial stem cell niches via single‐cell RNA sequencing (scRNA‐seq). The transcriptomic profiles and cellular compositions have been delineated, indicating alterations of the cranial bone marrow microenvironment influenced by inflammaging. Moreover, we identified a senescent mesenchymal cell subcluster and several age‐related immune cell subclusters by reclustering and pseudotime trajectory analysis, which might be closely linked to inflammaging. Finally, differentially expressed genes (DEGs) and cell–cell communications were analyzed during aging, revealing potential regulatory factors. Overall, this work highlights the age‐related changes in cranial stem cell niches, which deepens the current understanding of cranial bone and suture biology and may provide therapeutic targets for antiaging and regenerative medicine.

## Graphical abstract

This work investigated age‐related changes in murine cranial stem cell niches via scRNA‐seq. Transcriptomic profiles and cellular compositions were delineated, revealing alterations in the local milieu due to inflammaging. Besides, reclustering and pseudotime trajectory analysis unveiled a senescent mesenchymal cell subset and age‐related immune cell subsets potentially linked to inflammaging. DEGs and cell–cell crosstalks were further evaluated during aging, uncovering potential regulatory factors. Overall, this work highlights age‐related shifts in cranial stem cell niches, enhancing our grasp of cranial bone and suture biology, and suggests targets for antiaging and regenerative therapies.

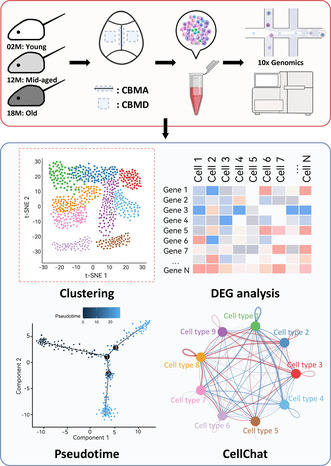

**Abbreviations**
CAR cell: CXCL12‐abundant reticular cell
CBMA: cranial bone marrow adjacent to the suture
CBMD: cranial bone marrow distant from the suture
cMo: classical monocyte
DC: dendritic cell
DEG: differentially expressed gene
DNT: double negative T cell
DPT: double‐positive T cell
ECM: extracellular matrix
FN1: fibronectin 1
GDT: γδ T cell
GO: gene ontology
GSEA: gene set enrichment analysis
GZMK: granzyme K
IGKC: immunoglobulin kappa constant
immNeu: immature neutrophil
iMo: intermediate monocyte
ISG: interferon‐stimulating gene
KEGG: Kyoto encyclopedia of genes and genomes
Macro: macrophage
MDP: monocyte‐macrophage/DC precursor
M‐MDSC: monocytic myeloid‐derived suppressor cell
mNeu: mature neutrophil
MNP: mononuclear phagocyte
MSC: mesenchymal stem cell
ncMo: non‐classical monocyte
NKT: natural killer T cell
NR4A1: nuclear receptor subfamily 4 group A member 1
Oc: osteoclast
OPN: osteopontin
OXPHOS: oxidative phosphorylation
pDC: plasmacytoid dendritic cell
PMN: polymorphonuclear neutrophil
PMN‐MDSC: polymorphonuclear myeloid‐derived suppressor cell
PTN: pleiotrophin
SASP: senescence‐associated secretory phenotype
SCLC: Schwann cell‐like cell
scRNA‐seq: single‐cell RNA sequencing
SDF‐1: stromal cell‐derived factor‐1
SSC: skeletal stem cell
STEM: short time‐series expression miner
SuSC: cranial suture mesenchymal stem cell
Taa: age‐associated T cell
Tex: exhausted T cell
TNC: tenascin c
Treg: regulatory T cell
UMAP: uniform manifold approximation and projection
VLA4: very late antigen 4
VSMC: vascular smooth muscle cell
ZFP36L2: zinc finger protein 36‐like 2

## 1 INTRODUCTION

Aging is a multifaceted and complicated process, manifested by the decline of normal physiological functions across tissues and organs, leading to overt frailty, mortality, and plenty of chronic diseases (Weng et al., 2022), which negatively affects the quality of life and health span. Notably, an overall decline of immunity often parallels the systemic aging process, affecting both the innate and adaptive immune systems. Counterintuitively, but in fact, immune cells undergo abnormal activation rather than suppression during aging, developing a persistent, chronic, low to mid‐grade inflammatory state, with compromised resistance to various pathological challenges, known as inflammaging (Almanan et al., 2020; Singh et al., 2019). In addition to systemic immune alterations, aging fundamentally impacts stem cells, a key player in maintaining homeostasis and responding to injury repair. Aging results in functional deterioration of stem cells, including permanent cell cycle arrest, impaired self‐renewal, and differentiation potentials, collectively referred to as stem cell senescence (Weng et al., 2022). Although the reciprocal interactions of stem cells and their niches remain unclarified, age‐related changes in stem cells are closely associated with inflammaging progression. Senescent stem cells typically secrete a combination of pro‐inflammatory factors, known as the senescence‐associated secretory phenotype (SASP; Khosla et al., 2022; Li et al., 2021), which plays a pivotal role in inflammaging progression via autocrine and/or paracrine. Besides, the aging stem cell niche skews hematopoietic differentiation toward the myeloid lineage, reinforcing cellular senescence and chronic inflammation (Tyrrell & Goldstein, 2021). Therefore, exploring the onset and progression of inflammaging from the perspective of stem cell niche is instrumental in comprehending both stem cell senescence and the broader phenomenon of systemic aging.

Upon aging, one of the most pronounced impairments occurs in the skeletal system, characterized by an impaired regeneration capacity. In the appendicular skeleton, intrinsic aging of skeletal stem cells (SSCs), also known as mesenchymal stromal or stem cells (MSCs), has been found to generate a degenerative inflammatory niche, leading to fragile bones that regenerate poorly (Ambrosi et al., 2021). Cranial bones are no exception. The self‐repair ability of cranial bones drastically decreases with age, leaving the organism vulnerable (Paige et al., 2006). In recent years, the cranial suture mesenchyme has been identified as a unique stem cell niche for homeostasis and tissue regeneration (Maruyama et al., 2016; Zhao et al., 2015). Indeed, suture mesenchyme is involved in a variety of physiological and/or pathological conditions, such as growth, injury repair, tissue remodeling, and craniosynostosis (Maruyama et al., 2021; Menon et al., 2021; Wilk et al., 2017; Yu et al., 2021; Zhao et al., 2015). Furthermore, deep imaging of murine calvaria via light‐sheet microscopy has revealed that SSCs and progenitor cells are spatially correlated with CD31^hi^Emcn^hi^ blood vessels, indicating both suture mesenchyme and cranial bone marrow serve as stem cell niches (Rindone et al., 2021). Thus, it is plausible to associate age‐related regenerative potential loss with alterations in cranial suture MSCs (SuSCs) and their niches. Recently, several groups have investigated the SuSCs or cranial suture biology in the context of craniosynostosis (Holmes et al., 2020), SSCs (Menon et al., 2021), healing (Aldawood et al., 2023; Xu et al., 2022), and embryonic development (Farmer et al., 2021; Holmes et al., 2021) utilizing single‐cell RNA sequencing (scRNA‐seq). However, none of the publications in this research area focused on age‐related changes in the cranial bone marrow, which also functions as a stem cell niche and deserves increased attention.

To this end, we aimed to delineate a comprehensive transcriptomic atlas to unravel age‐related changes in the two distinct cranial stem cell niches at single‐cell resolution. Also, we endeavor to gain valuable insights into the cellular and molecular alterations implicated in inflammaging. First, we depicted the immune cell landscape within cranial stem cell niches undergoing significant changes with age. Second, we identified several subclusters of immune cells closely linked to inflammaging, and a subset of senescent mesenchymal cells. Finally, mesenchymal cells were found to orchestrate cell–cell communication and modulate the local milieu. Overall, within the cranial stem cell niches, more pronounced transcriptional changes occur early rather than later in life; plus, early‐ and late‐life periods may not share the same gene regulatory networks. In conclusion, our study focused on the age‐related changes of cranial bone marrow and mesenchymal cells, which expands the current knowledge of cranial stem cell niches and craniofacial skeletal biology. Our data and findings may also enlighten regenerative medicine and anti‐senescence therapies.

## 2 RESULTS

### 2.1 Single‐cell atlas of different cranial stem cell niches by age and anatomic site

**FIGURE 1:**
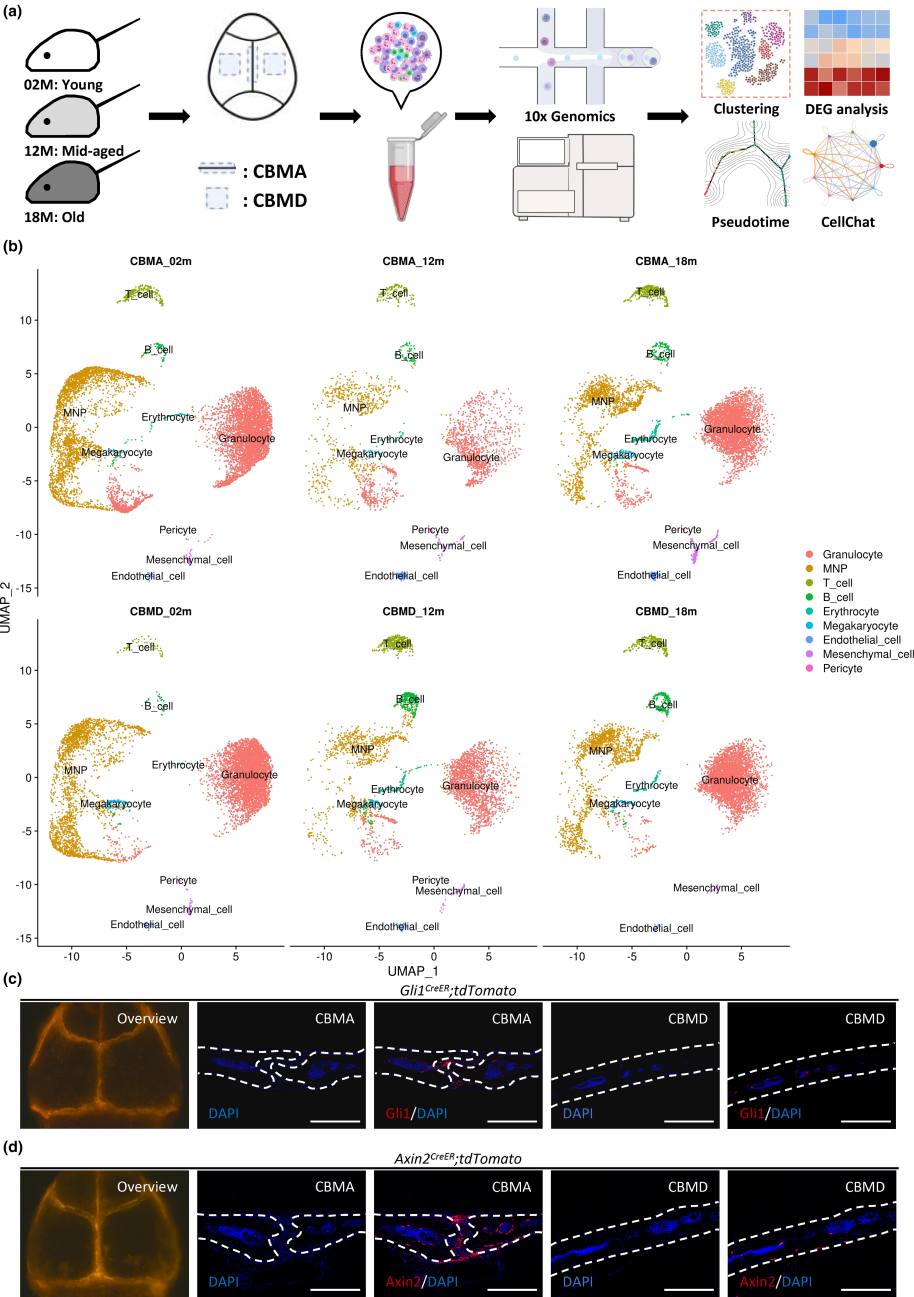
Overview of the workflow and the single‐cell atlas of cranial stem cell niches during aging. (a) Experimental design schematic. Briefly, six groups are established, including three groups of samples containing cranial bone marrow adjacent to the suture (CBMA_02m; CBMA_12m; CBMA_18m) and three groups of samples containing cranial bone marrow distant from the suture (CBMD_02m; CBMD_12m; CBMD_18m). All samples are processed using 10x Genomics Chromium platform. (b) UMAP method for dimensional reduction and visualization. UMAP plots segregated into CBMA_02m, CBMA_12m, CBMA_18m, CBMD_02m, CBMD_12m, and CBMD_18m groups. Cell types are clustered, annotated, and distinguished by colors. (c) Lineage tracing of Gli1+ SuSCs using *Gli1* ^*CreER*^; *tdTomato* mice induced with Tamoxifen. White dotted lines depict parietal bones and sagittal suture mesenchyme. Scale bars, 500 μm. (d) Lineage tracing of Axin2+ SuSCs using *Axin2* ^*CreER*^; *tdTomato* mice induced with tamoxifen. White dotted lines depict parietal bones and sagittal suture mesenchyme. Scale bars, 500 μm. Abbreviations: CBMA, cranial bone marrow (including the suture mesenchyme) adjacent to the suture; CBMD, cranial bone marrow distant from the suture; MNP, mononuclear phagocytes; SuSC, cranial suture mesenchymal stem cell; UMAP, Uniform Manifold Approximation and Projection.

To decipher the age‐related changes of cranial stem cell niches, that is suture mesenchyme and cranial bone marrow, we harvested tissues from wild‐type C57BL/6 mice for enzymatic digestion and performed scRNA‐seq on suspensions (Figure 1a). Three different age groups were set up: 2‐month‐old (02 m), 12‐month‐old (12 m), and 18‐month‐old (18 m). Each age group was then further subdivided based on the anatomic site of the harvested tissues. One group had the cranial bone marrow (including the suture mesenchyme) adjacent to the suture (CBMA), and the other group included the cranial bone marrow distant from the suture (CBMD). The scRNA‐seq libraries were generated using the 10× Genomics Chromium platform. After stringent quality control (QC; Figure S1A–C), six pooled samples with 35,894 cells were sequenced for subsequent analysis. Besides, we selected well‐established markers and conducted lineage tracing of Gli1+ (Zhao et al., 2015) and Axin2+ SuSCs (Maruyama et al., 2016) to confirm the different niches, CBMA and CBMD (Figure 1c,d).

**FIGURE 2:**
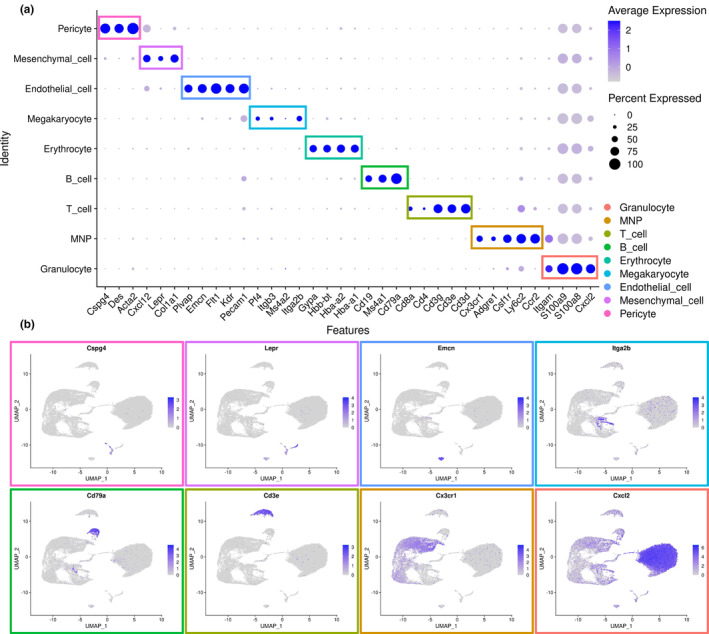
Cell‐type annotation and marker gene expression of cell clusters within cranial stem cell niches. (a) Dot plot demonstrating the expression of classical marker genes in different cell clusters. Cell types are distinguished by colors. (b) Feature plots demonstrating representative marker genes in different cell clusters. Cell types are distinguished by colors (erythrocyte cluster not shown).

Next, we applied the Uniform Manifold Approximation and Projection (UMAP) method for dimensional reduction and visualization. We identified a total of nine clusters, including granulocytes, mononuclear phagocytes (MNPs), T cells, B cells, erythrocytes, megakaryocytes, endothelial cells, mesenchymal cells, and pericytes, through unsupervised graph clustering (Figure 1b). The top 10 expressed genes of each cluster were presented (Figure S2A). Furthermore, all cell clusters were verified with several representative marker genes according to our recently published review (Li et al., 2023; Figures 2a,b, and S2B). Granulocytes, MNPs, and T cells were the top three most abundant cell types among cellular compartments, comprising over 80% of the total cells, which drove our focus on these immune clusters. Meanwhile, mesenchymal cells accounted for approximately 2.5% and 0.8% of all identified cells in CBMA and CBMD, respectively. Further detailed characterizations and subclustering analyses of these cell types were presented in subsequent sections.

### 2.2 Characterization of transcriptomic profiles and gene expression patterns of cranial stem cell niches

**FIGURE 3:**
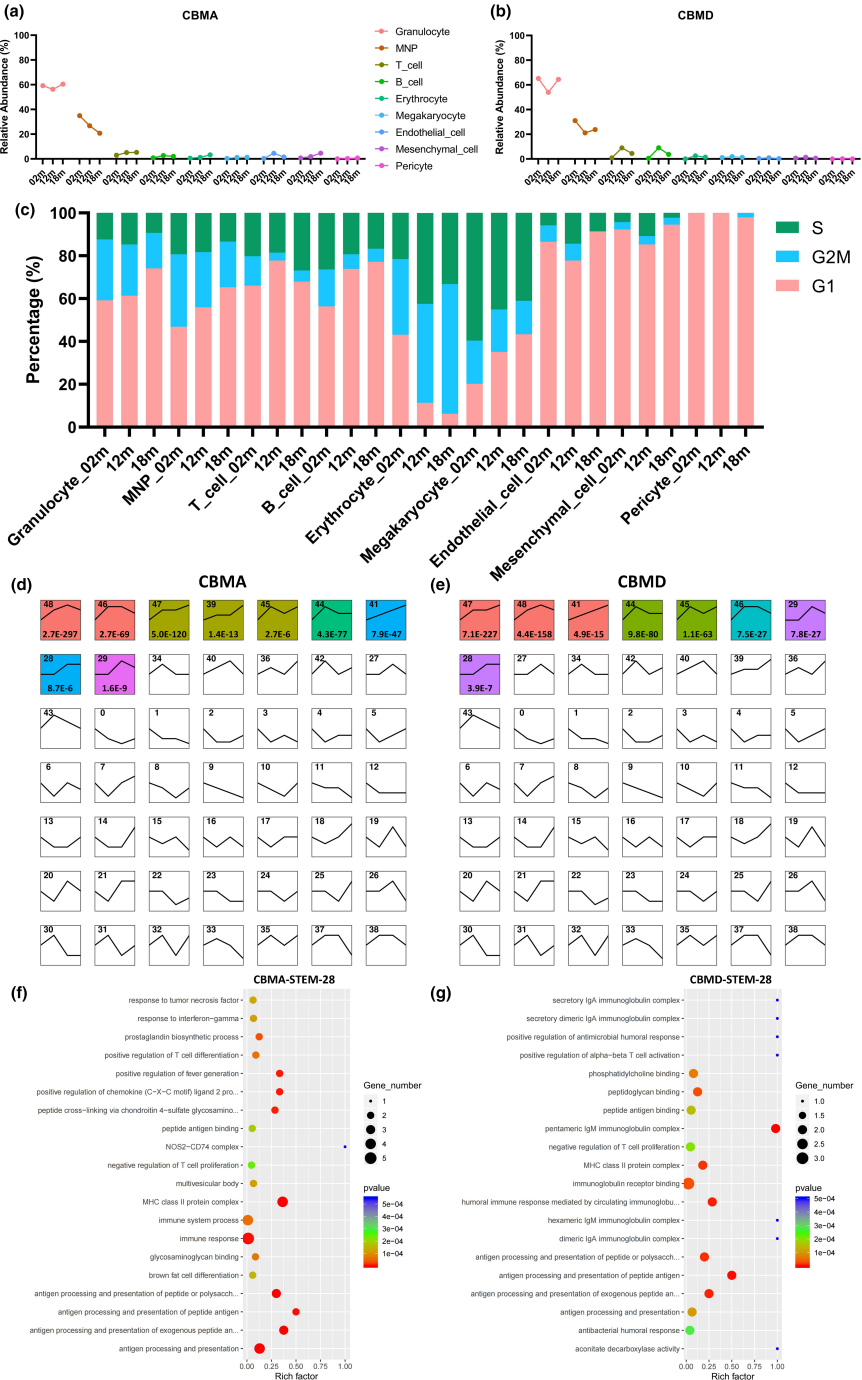
Characterization of the cellular composition, cell cycle distribution, and temporal gene expression profile. (a, b) Line charts depict the cellular composition of each cell type within CBMA (a) and CBMD (b) at different ages. Cell types are distinguished by colors. (c) Bar chart showing cell cycle distribution concerning each cell type within cranial stem cell niches (CBMA and CBMD) at different ages. Cell types are distinguished by colors. (d, e) STEM analysis showing temporal gene expression profiles in CBMA (d) and CBMD (e). Profiles enriched with statistical significance are color labeled with *p* values annotated. (f, g) GO analysis of genes related to profile 28 in CBMA (f) and CBMD (g). Top 20 enriched GO terms are displayed. Abbreviations: STEM, Short Time‐series Expression Miner; GO, Gene Ontology.

Six individual UMAP plots were displayed across age stages and anatomic sites (Figure 1b). Despite similarities between UMAP plots, the specific cell composition, particularly immune cells, varied by age and anatomical location (Figure S2C). Comparisons were made within the CBMA and CBMD groups, that is CBMA_02m versus CBMA_12m versus CBMA_18m and CBMD_02m versus CBMD_12m versus CBMD_18m, respectively, regarding the relative abundance of each cell type. For instance, in both the CBMA and CBMD groups, granulocyte counts initially decreased (12 m vs. 02 m) and later increased (18 m vs. 12 m) to a comparable level as the beginning (18 m vs. 02 m; Figure 3a,b). As for MNPs, in CBMA groups, MNP counts tended to decline along with aging (12 m vs. 02 m, 18 m vs. 12 m; Figure 3a); in CBMD groups, MNP counts first declined (12 m vs. 02 m), then elevated slightly (18 m vs. 12 m), but were still lower than baseline (18 m vs. 02 m; Figure 3b). As for T cells, in CBMA groups, their abundance was first enriched (12 m vs. 02 m) and then remained at a higher level with an almost unchanged percentage (18 m vs. 12 m, 18 m vs. 02 m; Figure 3a); in CBMD groups, the proportion of T cells was also drastically increased (12 m vs. 02 m), then reduced (18 m vs. 12 m), but still sustained a higher level than the beginning (18 m vs. 02 m; Figure 3b). Overall, it is difficult to conclude a general linear trend for age‐related changes in cranial stem cell niches.

Cell cycle arrest is a hallmark of aging and cellular senescence. Hence, we performed cell cycle analysis (Figure S2D). Not surprisingly, we found that most cell types underwent potential cell cycle arrest during aging, with higher portions of cells in the G1 phase and lower portions in the S and G2/M phases (Figure 3c). Next, we applied the Short Time‐series Expression Miner (STEM) software (Ernst & Bar‐Joseph, 2006) to analyze the transcriptomic changes over time. Our goal was to discover temporal gene expression profiles and identify the genes associated with these specific profiles (Figure 3d,e). As is shown, each small square represents a specific cluster containing a group of genes with the same expression profile (Figure 3d,e). In particular, we focused on the temporal expression profiles with statistical significance and upregulation tendency, including profiles 47, 39, 41, and 28 in CBMA (Figure 3d) and profiles 47, 41, and 28 in CBMD (Figure 3e). Since STEM is fully integrated with the Gene Ontology (GO) database, we performed gene enrichment analysis for the sets of genes involved in the above profiles. Eventually, we found enrichment in immune response, immune system process, antigen processing and presentation, inflammatory response, and regulation of immune cells, especially in profile 28 in both the CBMA and CBMD groups (Figure 3f,g), suggesting that inflammaging transcriptional alterations emerged as early as middle age (12 m) and persisted into old age (18 m). In addition, gene sets were also involved in protein binding, cytosol, cytoplasm, nucleus, regulation of transcription, and some other biological processes (Figure S3A–E).

### 2.3 Identification of senescent mesenchymal cells by subclustering and pseudotime trajectory analysis

**FIGURE 4:**
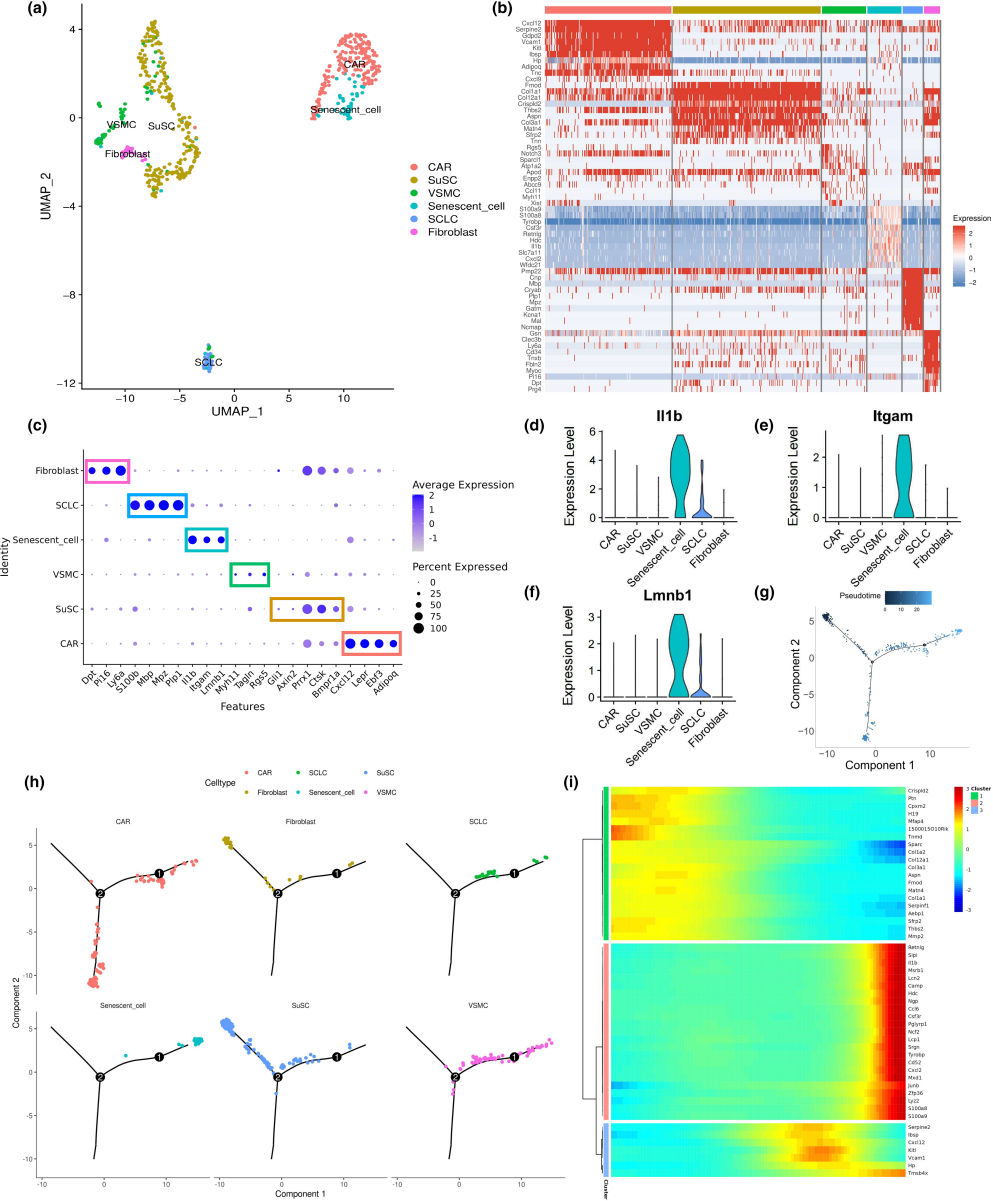
Subclustering and pseudotime trajectory analysis of the mesenchymal cell cluster. (a) UMAP plot of the reclustered mesenchymal cell population. Mesenchymal cell subsets are distinguished by colors. (b) Heatmap revealing six subsets of the mesenchymal cell cluster within cranial stem cell niches (CBMA and CBMD). Top 10 marker genes with the highest expression in each subset are displayed. (c) Dot plot demonstrating the expression of classical marker genes in different mesenchymal cell subsets. Mesenchymal cell subsets are distinguished by colors. (d–f) Violin plots depict expression levels of aging‐related marker genes, such as *Il1b* (d), *Itgam* (e), and *Lmnb1* (f), highly expressed by the senescent subset of mesenchymal cells. Mesenchymal cell subsets are distinguished by colors. (g) Developmental trajectory of mesenchymal cell subsets by pseudotime value. (h) Distribution of six mesenchymal cell subsets along the developmental trajectory. The senescent subset had the highest pseudotime value and was located at the ending point, whereas the SuSC subset had the lowest pseudotime value and was located at the starting point. (i) Clustered heatmap revealing top 50 genes with the most significant alterations across pseudotime in mesenchymal cell population. Abbreviations: CAR, CXCL12‐abundant reticular cell; SCLC, Schwann cell‐like cell; VSMC, vascular smooth muscle cell.

Recent studies have highlighted the heterogeneity and plasticity of mesenchymal cells (Tan et al., 2022; Wang, Chai, et al., 2021). Thus, from this perspective, we delved into the properties of cranial suture and bone marrow mesenchymal cells. Likewise, the cluster of mesenchymal cells in our study was identified according to the expression of SSC/MSC (*Lepr*, *Cxcl12*) and osteoblast (*Col1a1*) marker genes (Matsushita et al., 2020; Zhou et al., 2014). For in‐depth analysis, we reclustered the mesenchymal population and obtained six subclusters (Figure 4a). The subclusters were assigned identities based on the top 10 expressed genes using the Seurat function FindAllMarkers (Figure 4b) and validated by several classical marker genes (CAR [CXCL12‐abundant reticular cell], *Cxcl12*, *Lepr*, *Ebf3*, *Adipoq*; VSMC [vascular smooth muscle cell], *Myh11*, *Tagln*, *Rgs5*; senescent cell, *Il1b*, *Itgam*, *Lmnb1*; SuSC, *Gli1*, *Axin2*, *Prrx1*, *Ctsk*, *Bmp1ra*; SCLC [Schwann cell‐like cell], *S100b*, *Mbp*, *Mpz*, *Plp1*; Fibroblast, *Dpt*, *Pi16*, *Ly6a*; Figure 4c). Notably, we identified a unique subset of senescent mesenchymal cells exhibiting high expression of aging‐related genes (Figure 4c–f), whose senescence state was validated by using the Seurat function AddModuleScore, showing to possess the highest “senescent scores” (Figure S3F), herein referred to as senescent cells. However, some common senescence markers, such as *Cdkn2a* and *Cdkn1a*, were somehow not exclusively enriched in the core signature of the senescent subset, supporting the idea that known senescence markers lack universality across different cell types (Hernandez‐Segura et al., 2017).

To unveil age‐related changes in mesenchymal cells and properties of the senescent subset, we performed pseudotime analysis and reconstructed developmental trajectories using Monocle (Trapnell et al., 2014; Figure 4g). We found that six mesenchymal subclusters were distributed along branches of the pseudotime trajectory, with the senescent cell subcluster having the highest pseudotime value and positioned at the endpoint, indicating a terminal state of this senescent subset (Figure 4h). In contrast, SuSC had the lowest pseudotime value and was located at the beginning of the trajectory, indicating its role as a developmental origin for other mesenchymal cell subclusters (Figure 4h). In addition, we estimated RNA velocities in single cells using the velocyto.R package (La Manno et al., 2018) to assess transcriptional activity and kinetics (Figure S4A). Velocity fields and pseudotime values were projected onto the UMAP plot, providing insight into the origin and interrelationship of mesenchymal cell subsets, and we found great consistency between the UMAP plots in terms of cell differentiation status (Figure S4A,B). Therefore, we confirmed that the SuSC subset serves as the origin of mesenchymal cells while the senescent subset represents the terminal state, which potentially regulates inflammaging processes.

Furthermore, the top 50 genes with significant changes over pseudotime were clustered (Figure 4i). In general, many genes related to inflammatory processes showed an increased pattern, such as *Il1b*, *Cxcl2*, *S100a8*, and *S100a9* (Figures 4i and S4C). Intriguingly, typical SSC/MSC marker genes were also upregulated, such as *Cxcl12*, *Lepr*, *Ebf3*, and *Vcam1* (Figures 4i and S4D,E), indicating a self‐modulation of mesenchymal cells in response to inflammaging to preserve their stemness. Conversely, genes associated with fibrillar collagens and osteogenesis, such as *Col3a1*, *Col14a1*, *Col12a1*, *Col1a1*, and *Col1a2*, showed downregulation (Figures 4i and S4F), suggesting impaired osteogenic capacity in senescent cells. Further, differentially expressed genes (DEGs) analysis of mesenchymal cells validated the upregulation of *Cxcl12* and *Vcam1* and the downregulation of *Col1a1*, *Col3a1*, and *Col14a1* in both the CBMA and CBMD groups (Figure S5A–D).

### 2.4 Age‐related immune cell subsets changes within cranial suture and bone marrow microenvironment

Recent studies have highlighted the active involvement of immune cells, including T cells, MNPs, and granulocytes, in the establishment of a pro‐inflammatory niche (Almanan et al., 2020; Li et al., 2021; Mogilenko et al., 2021), which links inflammation to age‐related deterioration and may drive dysfunctional tissue changes. To investigate the role of immune cells, we performed a detailed analysis of T cells, granulocytes, and MNPs. By reclustering, we identified several subsets closely related to inflammaging and constitute an inflammatory milieu in cranial stem cell niches.

**FIGURE 5:**
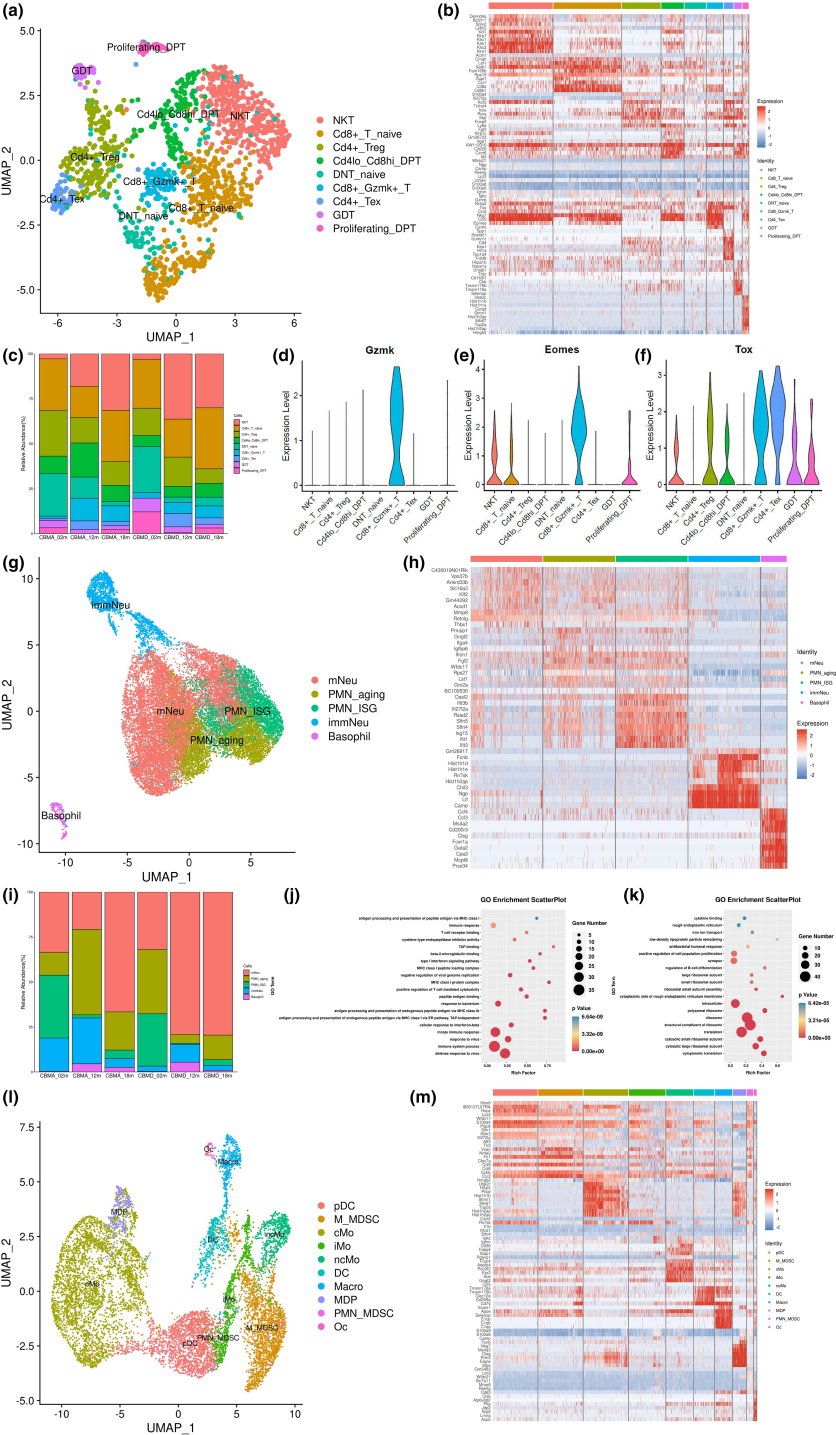
Subclustering and characterization of the immune cell clusters to reveal age‐related subsets. (a) UMAP plot of reclustered T‐cell population. T‐cell subsets are distinguished by colors. (b) Heatmap revealing nine subsets of the T‐cell cluster within cranial stem cell niches (CBMA and CBMD). Top 10 marker genes with the highest expression in each subset are displayed. (c) Bar chart showing cellular composition of T‐cell subsets within cranial suture and bone marrow at different ages. (d–f) Violin plots depict transcriptional signatures of Cd8 + Gzmk+ T‐cell subset, such as *Gzmk* (d), *Eomes* (e), and *Tox* (f). T‐cell subsets are distinguished by colors. (g) UMAP plot of reclustered granulocyte population. Granulocyte subsets are distinguished by colors. (h) Heatmap revealing five subsets of granulocyte cluster within cranial stem cell niches (CBMA and CBMD). Top 10 marker genes with the highest expression in each subset are displayed. (i) Bar chart showing cellular composition of granulocyte subsets within cranial suture and bone marrow at different ages. (j, k) GO analysis of marker genes related to PMN‐ISG subset (j) and PMN‐aging subset (k). Top 20 enriched GO terms are displayed. (l) UMAP plot of reclustered MNP population. MNP subsets are distinguished by colors. (m) Heatmap revealing 10 subsets of MNP cluster within cranial stem cell niches (CBMA and CBMD). Top 10 marker genes with the highest expression in each subset are displayed. Abbreviations: cMo, classical monocyte; DC, dendritic cell; DNT, double‐negative T cell; DPT, double‐positive T cell; GDT, γδ T cell; immNeu, immature neutrophil; iMo, intermediate monocyte; ISG, interferon‐stimulating gene; Macro, macrophage; MDP, monocyte–macrophage/dendritic cell precursor; M‐MDSC, monocytic myeloid‐derived suppressor cell; mNeu, mature neutrophil; ncMo, nonclassical monocyte; NKT, natural killer T cell; Oc, osteoclast; pDC, plasmacytoid dendritic cell; PMN, polymorphonuclear neutrophil; PMN‐MDSC, polymorphonuclear myeloid‐derived suppressor cell; T naive, naïve T cell; Tex, exhausted T cell; Treg, regulatory T cell.

Several T‐cell subclusters were identified based on marker gene expression, including double‐negative T cell (DNT), γδ T cell (GDT), natural killer T cell (NKT), naïve T cell (T_naive), double‐positive T cell (DPT), exhausted T cell (Tex), and regulatory T cell (Treg; Figure 5a,b). When comparing CBMA and CBMD, we found a similar alteration in the cellular compartments. Specifically, naïve DNT (DNT_naive) and Treg decreased with age, whereas NKT, GDT, and Cd8 + Gzmk+ T cells increased (Figure 5c). Among increased subclusters, we focused on a so‐called “Cd8+Gzmk+ T cell” subcluster, named for exclusively expressing the *Gzmk* transcript (Figure 5d), which encoded granzyme K (GZMK) and culminated in inducing apoptosis. This subset was enriched in the middle‐aged (12 m) and elderly (18 m) groups and less abundant in the young (02 m) groups (Figure 5c). Moreover, Cd8 + Gzmk+ T cells possessed distinct transcriptional signatures with high expression of Tex marker genes, such as *Eomes* and *Tox* (Figure 5e,f). Interestingly, these Cd8 + Gzmk+ T cells resembled a population termed “age‐associated T cell (Taa)” in a recent immunological study (Mogilenko et al., 2021). Taa has been validated as a conserved hallmark of inflammaging in mice and humans. Due to the aging host environment, they accumulate in tissues and continuously secrete GZMK, promoting SASP from senescent cells (Mogilenko et al., 2021). Hence, it can be hypothesized that Cd8 + Gzmk+ T cells found in our study may function similarly to their counterpart Taa and enhance the inflammatory functions of non‐immune cells, resulting in an unfavorable microenvironment for resident mesenchymal cells.

We also analyzed granulocyte subclusters and identified basophil, immature neutrophil (immNeu), mature neutrophil (mNeu), and polymorphonuclear neutrophil (PMN; Figure 5g). PMN could be divided into two subclusters according to their transcriptional signatures (Figures 5h and S6A,B). One PMN subset preferentially expressed interferon‐stimulating genes (ISGs), such as *Ifit1*, *Ifit3*, and *Isg15* (Figures 5h and S6A), and appeared similar to the population of “PMNb (G5b‐ISG)” described by Xie et al. (2020); herein we refer to “PMN‐ISG”. The other PMN subset was similar to the population of “PMNc (G5c)” (Xie et al., 2020), which appeared to be more aged, so we refer to it as “PMN‐aging” (Figures 5h and S6B). Notably, PMNb (G5b‐ISG) was reported to expand during bacterial infection (Xie et al., 2020), and we found that the proportion of PMN‐ISG decreased drastically during aging (18 m vs. 02 m; Figure 5i). We then performed GO analysis of PMN‐ISG and found significant enrichment in immune response, immune system process, antigen processing and presentation, and response to virus and bacterium (Figure 5j), suggesting attenuated immune response with aging. The PMN‐aging subset was enriched in CBMA_12m and CBMD_02m rather than in 18 m groups (Figure 5i), indicating that cellular senescence occurred independently of systematic aging. In addition, GO analysis revealed that PMN‐aging marker genes were enriched for translation and ribosome biogenesis (Figure 5k), implying a potentially high functionality of this subset.

Further, we investigated MNP subsets; in total, 10 subclusters were identified, including dendritic cell (DC), plasmacytoid DC (pDC), monocytic myeloid‐derived suppressor cell (M_MDSC), polymorphonuclear MDSC (PMN_MDSC), macrophage (Macro), monocyte–macrophage/DC precursor (MDP), osteoclast (Oc), classical monocyte (cMo), intermediate monocyte (iMo), and nonclassical monocyte (ncMo; Figure 5l,m). First, we compared the cellular composition of MNP subsets of different groups and determined that M‐MDSC might be a major player during aging. For one reason, the portion of M‐MDSC significantly enriched with aging in both CBMA and CBMD (Figure S6C), with marker genes *Itgam*, *Ccr2*, and *Ly6c2* highly expressed and meanwhile negative for *Ly6g* (Figure S6D–G). For another reason, MDSCs have been well known for their potent immunosuppressive function and negatively regulate immune responses in various conditions, such as cancer, chronic inflammation, and autoimmune diseases (Bronte et al., 2016; Veglia et al., 2018, 2021). Since aging is usually associated with chronic inflammation and aberrant myelopoiesis (Bronte et al., 2016; Dorshkind et al., 2020), we reasoned that cell cycle abnormalities might exist in myeloid cells. Hence, we performed cell cycle analysis of MNP subsets and projected cell cycle phases onto their UMAP plot. Interestingly, except cMo and MDP, most MNP subsets were in the G1 phase (Figure S6H), which might explain the accumulation of immature myeloid cells and/or MDSCs and also indicate the occurrence of inflammaging within the cranial stem cell niches.

### 2.5 Functional analysis of DEGs and screening for common upregulated DEGs at sample and cluster levels

To uncover changes in signaling pathways during aging, we conducted the gene set enrichment analysis (GSEA) (Subramanian et al., 2005) using the Kyoto Encyclopedia of Genes and Genomes (KEGG) (Kanehisa et al., 2021) pathway database against age‐related DEGs (18 m vs. 02 m). As a result, 10 out of the top 15 KEGG terms enriched in the CBMA and CBMD groups were shared, implying similar age‐related transcriptomic changes in cranial stem cell niches. Among these common terms, four terms were positively correlated with cytokine and cytokine receptor interaction, T‐cell receptor signaling pathway, pathways in cancer, and MAPK signaling pathway (Figure S7A–H); six terms showed a negative correlation with oxidative phosphorylation (OXPHOS), proteasome, ribosome, and, interestingly, several neurodegenerative disorders, including Huntington's disease, Alzheimer's disease, and Parkinson's disease (Figure S7I–T). Align with previous studies, widespread decreases in OXPHOS, proteasomal degradation, and reduced ribosome production are commonly observed in aging (Mattson & Arumugam, 2018; Nakamura‐Ishizu et al., 2020); moreover, the functioning of ribosomes and proteasomes are interconnected (Kampen et al., 2021); therefore, these three KEGG terms negatively correlated with aging were reasonable. However, the other three KEGG terms related to neurodegenerative diseases were more obscure, which might indicate a compensatory mechanism for age‐associated diseases. The CBMA and CBMD groups also had unique KEGG terms (Figure S7U–X). For instance, age‐related DEGs of CBMA were enriched in focal adhesion and extracellular matrix (ECM) receptor interaction (Figure S7U,V), which might relate to the structure of the suture complex. By contrast, age‐related DEGs of CBMD showed a negative correlation with DNA replication (Figure S7W) and a positive correlation with the neurotrophin signaling pathway (Figure S7X), again implying compensatory events for nerve degeneration.

**FIGURE 6:**
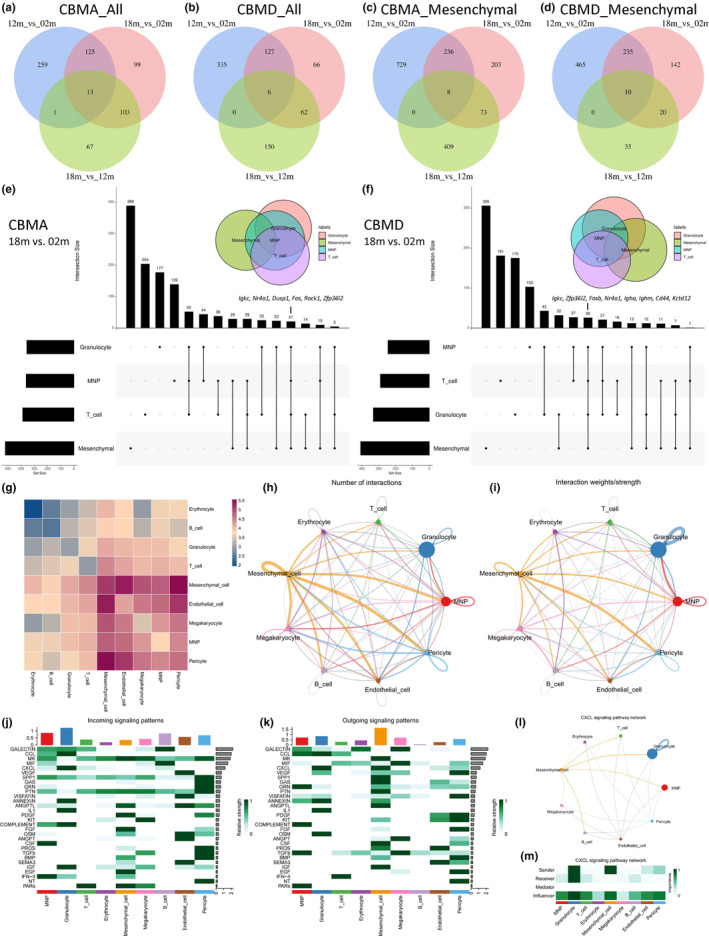
Integrated comparative analysis of shared upregulated DEGs and cell–cell crosstalk within cranial stem cell niches. (a–d) Venn diagrams showing overlaps of age‐related upregulated DEGs of pairwise comparisons among different ages. The upregulated DEGs are screened out from all cell clusters of CBMA (a) and CBMD (b); or the mesenchymal cell cluster of CBMA (c) and CBMD (d). (e, f) Upset plots demonstrating upregulated DEGs in the mesenchymal cell, T cell, granulocyte, and MNP clusters between CBMA_18m and CBMA_02m (e) or between CBMD_18m and CBMD_02m (f). (g) Heatmap revealing the relative cell–cell interaction number among different cell clusters within cranial stem cell niches, analyzed using CellPhoneDB. (h, i) Circle plots demonstrating the cell–cell interaction number (h) and weights/strength (i) of different cell clusters within cranial stem cell niches, analyzed using CellChat. (j, k) Heatmap revealing the incoming (j) and outgoing (k) signaling patterns of different cell clusters within cranial stem cell niches, analyzed using CellChat. Top 30 most significant signaling pathways are displayed. (l, m) Circle plot depicts inferred CXCL signaling networks within cranial stem cell niches (l), and the signaling roles of different cell clusters are revealed by heatmap (m), analyzed using CellChat. Abbreviations: DEG, differentially expressed genes.

To further unveil age‐related transcriptomic changes, we screened out DEGs between different age groups (12 m vs. 02 m, 18 m vs. 02 m, 18 m vs. 12 m) in CBMA and CBMD (Figure 6a,b), and focused on upregulated DEGs in mesenchymal cell, T cell, granulocyte, and MNP clusters (Figures 6c,d, and S8A–F). In general, among all comparisons between varied age groups, 12 m versus 02 m had the highest number of upregulated DEGs, followed by 18 m versus 02 m. In contrast, 18 m versus 12 m had the lowest number of upregulated DEGs (Figures 6a–d and S8A–F). Thus, massive transcriptional changes occurred early in life (from 02 to 12 m) and declined to a lesser extent later in life (from 12 to 18 m). Moreover, the overlaps between upregulated DEGs in different age groups (12 m vs. 02 m, 18 m vs. 02 m, 18 m vs. 12 m) were also assessed by Venn diagrams (Figures 6a–d and S8A–F). As a result, the overlap between 12 m versus 02 m and 18 m versus 02 m had the highest number of shared DEGs, suggesting early‐life (from 02 m to 12 m) transcriptomic changes have great similarities with the whole‐life (from 02 m to 18 m) transcriptomic changes; 18 m versus 02 m and 18 m versus 12 m had a moderate number of common DEGs; by contrast, 18 m versus 12 m and 12 m versus 02 m had minimal or no overlapping DEGs, implying late life (from 12 m to 18 m) and early life (from 02 m to 12 m) may involve distinct gene regulatory networks.

In addition, we applied upset plots (Conway et al., 2017) to depict age‐related transcriptional alterations among the four virtual clusters (mesenchymal cells, T cells, granulocytes, and MNPs; Figures 6e,f, and S8G–J). The mesenchymal cell cluster always had the highest number of upregulated DEGs (Figures 6e,f, and S8G–J), suggesting its plasticity and dynamic changes during aging, while the granulocyte cluster usually had the lowest number of upregulated DEGs (Figures 6e,f, and S8G–J). As aforementioned, early‐ and whole‐life comparisons showed the highest similarities regarding transcriptomic changes. Therefore, we focused on the intersections of upregulated DEGs of the four vital clusters in early‐ (12 m vs. 02 m) and whole‐life (18 m vs. 02 m) comparisons. Our upset plots proposed several candidate DEGs functioning in regulating the immune milieu during aging that were upregulated in both CBMA and CBMD (Figures 6e,f and S8G,H). These DEGs were *Igkc*, encoding immunoglobulin kappa constant (IGKC), with GO annotations including antigen binding and immune response; *Zfp36l2*, encoding zinc finger protein 36‐like 2 (ZFP36L2), an RNA‐binding protein, which has been reported to suppress terminal myeloid cell differentiation (Wang, Zhou, et al., 2021) and Treg function (Makita et al., 2020); *Nr4a1*, encoding nuclear receptor subfamily 4 group A member 1 (NR4A1), a proven key mediator of T‐cell dysfunction (Liu et al., 2019).

### 2.6 Age‐related changes in mesenchymal–immune cell crosstalk in different cranial stem cell niches

Intimate crosstalk between stem cells and their supportive niches is pivotal for proper stem cell maintenance and cell fate commitment (Fuchs & Blau, 2020). Thus, we employed CellPhoneDB (Efremova et al., 2020) and CellChat (Jin et al., 2021) to decipher intercellular communications, which enabled us to visualize and quantify cell–cell crosstalk based on the expression levels of ligand–receptor pairings. Consequently, various cell populations engaged in extensive crosstalk. Within the cranial stem cell niches, we found that the mesenchymal cell population played a central role in orchestrating intercellular communications and microenvironment modulations, as supported by the number and weights/strength of interactions, which far exceeded other cell types (Figure 6g–i).

**FIGURE 7:**
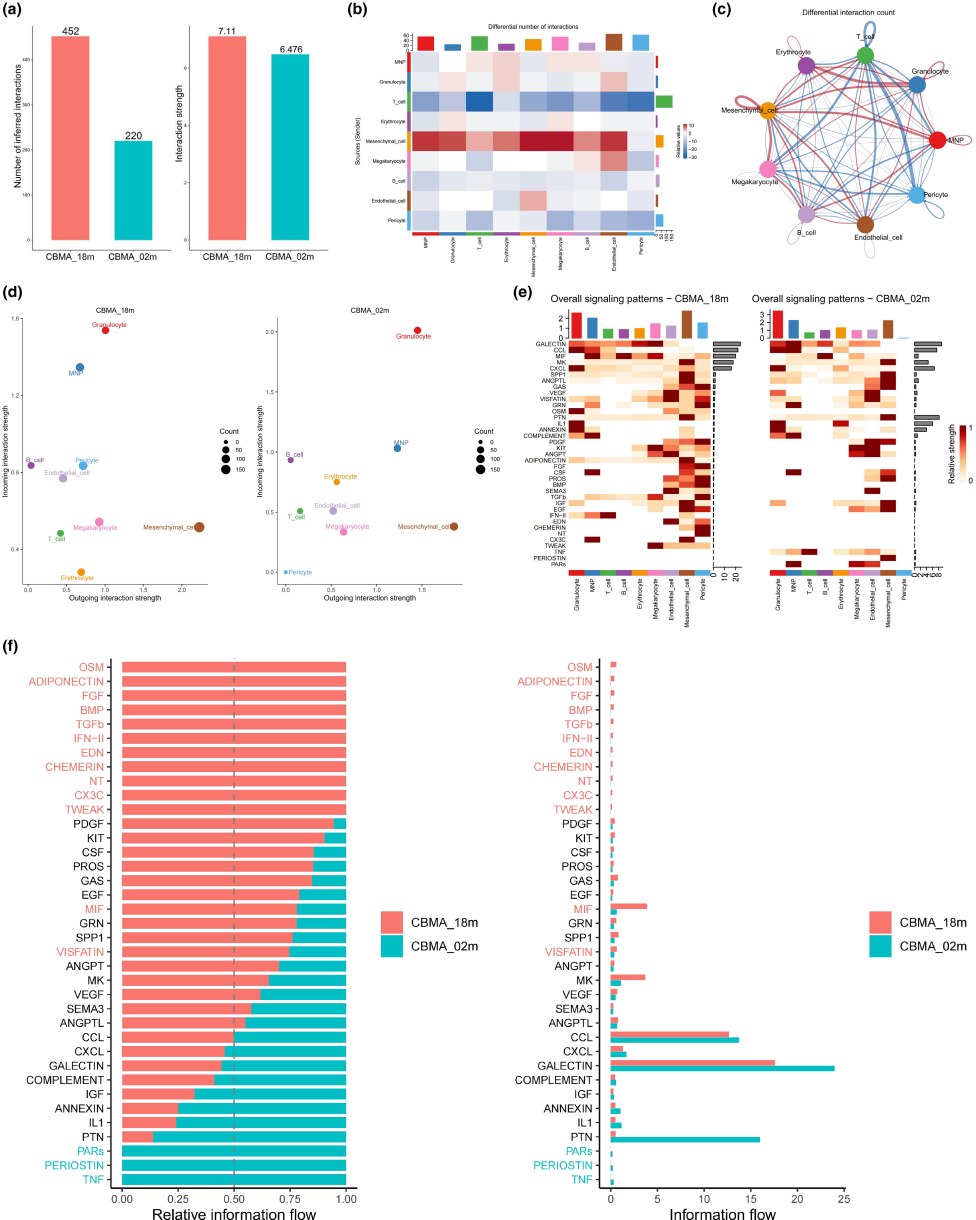
Age‐related changes of intercellular communications in CBMA. (a) Bar chart showing the overall differences of interaction number (left) and strength (right) between CBMA_18m and CBMA_02m, analyzed using CellChat. (b and c) Heatmap (b) and circle plot (c) presented differential interaction numbers among different cell clusters within CBMA, comparing CBMA_18m with CBMA_02m. (d) Scatter plots demonstrating the incoming and outgoing interaction strength of different cell clusters of CBMA_18m (left) and CBMA_02m (right), analyzed using CellChat. (e) Heatmaps revealing overall signaling patterns of different cell clusters of CBMA_18m (left) and CBMA_02m (right), analyzed using CellChat. The darker the color, the stronger the relative interaction strength. (f) Bar plots display the relative information flow (left) and information flow comparisons (right) of differential signaling pathways between CBMA_18m and CBMA_02m.

Our CellPhoneDB analysis revealed the top 20 ligand–receptor pairings (Figure S9A). Among these pairings, VCAM1‐α4β1 integrin (aka very late antigen 4, VLA4), TNC (tenascin C)‐α4β1 integrin (VLA4), SPP1 (aka osteopontin, OPN)‐α4β1 integrin (VLA4), FN1 (fibronectin 1)‐α4β1 integrin (VLA4), COL4A1‐α1β1 integrin (VLA1), and CXCL12 (aka stromal cell‐derived factor‐1, SDF‐1)‐CXCR4 are primarily involved in the intercellular crosstalk of the mesenchymal cells, especially the CXCL12–CXCR4 axis (Figure S9A). Furthermore, the CellChat analysis revealed the major signaling sources and targets, suggesting that the mesenchymal cell cluster serves as a sender/receiver (primarily as a sender) and coordinates with various signaling pathways, such as CXCL, SPP1, ANGPTL (angiopoietin like proteins), and PTN (pleiotrophin), to drive mesenchymalimmune cell crosstalk (Figures 6j–m and S9B–F). Next, to decipher age‐related changes in cell‐to‐cell communications in different cranial stem cell niches, we compared CBMA_18m with CBMA_02m (Figure 7a–f) and CBMD_18m with CBMD_02m (Figure S10A–F). We found that the number of interactions increased during aging in CBMA and CBMD (Figures 7a and S10A); the interaction strength increased in CBMA but declined in CBMD (Figures 7a and S10A). Thus, both the number and strength of interactions dramatically change with age.

The number of differential interactions of each cluster was also analyzed (Figures 7b,c and S10B,C). During aging, the mesenchymal cell cluster had the most remarkable increase in differential interactions, again indicating its pivotal role in the complicated intercellular communication network, when comparing CBMA_18m with CBMA_02m (Figure 7b,c) and CBMD_18m with CBMD_02m (Figure S10B,C). In contrast, the T cells in CBMA and the erythrocytes in CBMD showed the most drastic decrease in differential interactions during aging (Figures 7b,c and S10B,C). Regarding the signaling role of each cell cluster, all clusters maintained a similar status during aging when comparing CBMA_18m with CBMA_02m (Figure 7d) and CBMD_18m with CBMD_02m (Figure S10D). Finally, according to the overall signaling patterns and relative information flow, we suggest the following signaling pathways are most likely implicated in regulating the cranial stem cell niche during aging, including the upregulated signaling, such as CCL, CXCL, MIF, SPP1, and ADIPONECTIN, and the downregulated signaling, such as PERIOSTIN, MK, PTN, and GALECTIN, due to their differential expression with age change (Figures 7e,f and S10E,F).

## 3 DISCUSSION

Over the past decades, the origin and specific niche of the major stem cell population within long bones have been extensively studied, revealing not only the biological properties of SSCs/MSCs but also their typical perivascular niche within the bone marrow (Chan et al., 2015, 2018). Contrariwise, we have limited knowledge concerning the resident stem cells and their niches within craniofacial bones. Only in recent years, the suture mesenchyme has been identified as a unique niche for SSCs/MSCs in craniofacial bones (Maruyama et al., 2016; Zhao et al., 2015). Besides cranial bone marrow, the suture mesenchyme possesses robust regenerative capacity and contributes to homeostasis (Park et al., 2016). Nevertheless, a comprehensive comparison and investigation of the different cranial stem cell niches, including age‐related changes in resident SSCs/MSCs and their niches, are yet to be undertaken. These knowledge gaps hinder our understanding of the mechanisms underlying poor calvarial regeneration and increased vulnerability with aging.

Our study investigated the dynamic changes in cranial stem cell niches during aging. We demonstrated significant alterations in cellular compositions and transcriptomic profiles, contributing to the inflammaging phenotype. Nonuniform changes are observed among different age and anatomical location groups, particularly in immune cells. Intriguingly, transcriptional changes occur more radically in early life than in later stages within the local milieu, also implying that transcriptional signatures of inflammaging in cranial stem cell niches might emerge early in life. If so, timely implementation of targeted interventions could improve age‐related changes and promote tissue rejuvenation. However, it should be noted that our scRNA‐seq data are cross‐sectional and collected at different time points (02, 12, and 18 m). Consequently, individual animals or cells cannot be studied longitudinally due to the experimental design. Therefore, the reported findings on transcriptional changes over time are also cross‐sectional, generated from different animal donors at varying time points rather than being longitudinal in nature (Uyar et al., 2020). Nonetheless, such limitation of the single‐cell studies might be overcome by “Live‐seq,” a technique enabling the temporal transcriptomic profiling of single cells (Chen et al., 2022).

Furthermore, assessing MSCs/SSCs or immune cells within human cranial bones using scRNA‐seq faces significant challenges arising from various factors, including ethical considerations. In such scenarios, alternative strategies may be essential to derive more meaningful data. One approach involves utilizing publicly available human data resources for mining and analysis, such as the UK Biobank. Meanwhile, several cutting‐edge technologies are under development, aiming to characterize and unravel spatial proteomics and/or spatial transcriptomics in the region of interest. These advances may provide valuable temporospatial information about the intricate cellular and molecular components in heterogeneous tissues, thus aiding in the comprehensive understanding of cranial bones and sutures. The integration of these complementary approaches has the potential to exponentially enhance our current knowledge in this field and contribute to the progress of cranial bone and suture research in clinical contexts (Li et al., 2023).

Previous studies have linked immune cells to cellular senescence and inflammaging (Almanan et al., 2020; Khosla et al., 2022; Li et al., 2021; Mogilenko et al., 2021). Likewise, we uncovered the proportional changes of immune cells at the cluster and subcluster levels. Our scRNA‐seq datasets also identified several immune cell subsets closely associated with chronic inflammation and aging. Specifically, we found a Cd8 + Gzmk+ T‐cell subset in the T‐cell cluster, PMN‐ISG and PMN‐aging subsets in the granulocyte cluster, and M‐MDSC in the MNP cluster, which highly resembled the observations reported by other groups (Bronte et al., 2016; Mogilenko et al., 2021; Xie et al., 2020). Although we did not identify new immune cell subsets, it is essential to note that age‐related immune cell changes in the cranial bone marrow may have tissue‐specific features. Unlike other bone marrow regions, the cranial bone marrow remains hematopoietically active throughout life (Poller et al., 2020) and serves as a myeloid cell reservoir (Cugurra et al., 2021; Herisson et al., 2018). Further exploration of the cranial bone marrow from this perspective would be an intriguing avenue of research.

Intercellular crosstalk within the stem cell niche is essential for stem cell function and fate decisions (Fuchs & Blau, 2020). Our CellPhoneDB and CellChat analyses revealed that the mesenchymal cell cluster had the most active cell–cell communication among other cell types within the niches, as evidenced by the number and weight of interactions. Thus, the mesenchymal cell population is central in orchestrating intercellular crosstalk and modulating the immune microenvironment. Several signaling pathways, such as CCL, CXCL, MIF, SPP1, ADIPONECTIN, PERIOSTIN, MK, PTN, and GALECTIN, were implicated in regulating aged cranial stem cell niches according to our CellChat analysis, which revealed their differential expression levels during aging. Meanwhile, based on CellPhoneDB analysis, the CXCL12–CXCR4 axis and the VCAM1–VLA4 axis may play significant roles and correlate with each other, as CXCL12 signaling through CXCR4 activation has been reported to augment cell adhesion to VLA4 via VCAM1 (Petty et al., 2009). Taken together, the CXCL12–CXCR4 axis and the SPP1 signaling may be fundamental mechanisms regulating the cranial stem cell niche, although further experimental confirmation is needed.

## 4 CONCLUSION

To conclude, we presented a single‐cell transcriptomic atlas of cranial stem cell niches differentiated by age and anatomic site. Our study revealed multiple attributes of suture mesenchyme and cranial bone marrow microenvironment during aging progression, including gene expression patterns, mesenchymal and immune cell subsets changes, and cell–cell communications, which may deepen the current understanding of cranial bone biology and have potential implications for regenerative medicine and antiaging therapies.

## 5 MATERIALS AND METHODS

### 5.1 Animal experiments

The following mice were purchased from Jackson Laboratory: *Gli1* ^*CreER*^ (JAX007913), *Axin2* ^*CreER*^ (JAX018867), and *tdTomato* ^*Ai14*^ (JAX007914). *Gli1* ^*CreER*^; *tdTomato* and *Axin2* ^*CreER*^; *tdTomato* mice were generated and injected intraperitoneally (i.p.) at postnatal Day 30 (P30) with tamoxifen (Sigma‐Aldrich, T5648‐1 g) at a dosage of 0.25 g/kg body weight on P30, P32, and P34. Wild‐type C57BL/6 mice were purchased from GemPharmatech Co., Ltd. All animal experiments were carried out in accordance with the guidelines of the Institutional Animal Care and Use Committee (IACUC) at the State Key Laboratory of Oral Diseases, West China Hospital of Stomatology, Sichuan University (WCHSIRB‐D‐2022‐274).

### 5.2 Tissue dissection and preparation of single‐cell suspensions

Mouse cranial bone marrow tissue was harvested from wild‐type C57BL/6 mice of three different age groups, that is two months old (02 m), twelve months old (12 m), and eighteen months old (18 m), with six to eight male mice in each group. In total, six libraries were created, including three libraries of cranial bone marrow adjacent to the suture (CBMA_02m; CBMA_12m; CBMA_18m) and three libraries of cranial bone marrow distant from the suture (CBMD_02m; CBMD_12m; CBMD_18m).

To isolate the cranial bone marrow, the entire calvarium was harvested and processed to prepare cranial bone marrow single‐cell suspensions, which was adopted from a peer‐reviewed high‐profile publication (Cugurra et al., 2021). In brief, the calvarium was dissected under a stereomicroscope, with the removal of dura mater and periosteum, and transferred into a 60 mm RNase‐free culture dish on ice, containing an appropriate amount of calcium‐ and magnesium‐free 1× PBS (0.04% BSA). The sagittal sutures and adjacent parietal bones were collected for CBMA groups; meanwhile, the parietal bones distant from cranial sutures were collected for CBMD groups. The collected cranial tissues were then cut into 0.5 mm^2^ pieces using sterile scissors and mechanically dissociated in 1× PBS (0.04% BSA) with a pestle, followed by enzymatic digestion (1 mg/mL collagenase I + 3 mg/mL collagenase II + 2 mg/mL dispase II in 10 mL MEMα) for 15 min at 37°C with brief vortexing every 5 min. The digestion process was terminated with 3 mL MEMα containing 10% FBS and then pipetting 5–10 times.

The resulting cell suspension was filtered through a 70‐μm cell strainer and centrifuged at 420 ***g*** for 4 min at 4°C. The cell pellet was resuspended in 100 μL 1× PBS (0.04% BSA) and added with 1 mL 1× Red Blood Cell Lysis Solution (Miltenyi Biotec, 130‐094‐183) and incubated on ice for 5–10 min. After incubation, the suspension was centrifuged at 420 ***g*** for 4 min at 4°C and processed to remove dead cells using MACS® Dead Cell Removal Kit (Miltenyi Biotec, 130‐090‐101), followed by washing in 1× PBS (0.04% BSA) and centrifuging at 420 ***g*** for 4 min at 4°C. The cell pellet was resuspended in 50 μL of 1× PBS (0.04% BSA). The overall cell viability was confirmed by trypan blue exclusion assay (Thermo Fisher Scientific, SV3008401), which was not less than 85%. Finally, the concentration of single‐cell suspensions was determined using the automated cell counter (Invitrogen, Countess II) and adjusted to 700–1200 cells/μL.

### 5.3 Library preparation and sequencing

The CBMA and CBMD single‐cell suspensions were loaded to 10× Chromium single‐cell platform (10× Genomics) to capture around 5000 single cells, which was performed with the 10× Chromium Single Cell 3’ Reagent Kit v3 (10× Genomics, 1000092) according to the manufacturer's instructions. Next, the cDNA amplification and library construction steps were conducted according to the standard protocol. Libraries were paired‐end sequenced on a NovaSeq 6000 Sequencing System (Illumina) by LC‐Bio Technology Co. Ltd. at a minimum depth of 20,000 reads per cell.

### 5.4 Preprocessing of scRNA‐seq data and QC

Sequencing data were demultiplexed and converted to FASTQ format using bcl2fastq software (Illumina, version 2.20). The Cell Ranger Single‐Cell Software Suite (version 5.0.1) was used to perform sample demultiplexing, barcode processing, and single‐cell 3′ gene counting. All scRNA‐seq data were aligned to the mouse reference genome GRCm38 from Ensembl. The Cell Ranger output was loaded into the Seurat software (version 3.1.1) for dimensional reduction, clustering, and analysis of scRNA‐seq data. Overall, 35,894 cells passed the QC threshold: all genes expressed in more than three cells (default parameters: 1 cell); 500 genes expressed per cell as low cut‐off; 5000 genes expressed per cell as high cut‐off; UMI counts more than 500; the percentage of mitochondrial genes less than 25%.

### 5.5 Dimension reduction and clustering

To visualize the data, we further reduced the dimensionality of all 35,894 cells using Seurat software and utilized UMAP to project the cells into 2D space. In detail, normalization of the gene expression value was performed using the “NormalizeData” function and the built‐in LogNormalize method of the Seurat software. Next, principal component analysis (PCA) was performed using the normalized gene expression value. Within all the principal components (PCs), the top 10 PCs were used to do clustering and UMAP analysis. A graph‐based clustering method was applied to identify clusters, which relied on a clustering algorithm based on weighted shared nearest neighbor (SNN) modularity optimization. Marker genes for each cluster were identified with the Wilcoxon rank‐sum test with default parameters via the “FindAllMarkers” function of the Seurat software. The selected marker genes were expressed in more than 10% of the cells in a cluster with an average log fold change (logFC) above 0.25.

### 5.6 Gene functional annotation

KEGG pathway analyses and GO annotations were performed using the R package clusterProfiler (version 4.0) (Wu et al., 2021; Yu et al., 2012), and the software package is available at https://github.com/YuLab‐SMU/clusterProfiler.

GSEA was performed using the curated gene sets of Canonical Pathways and Hallmarks from MSigDB database (Liberzon et al., 2011) to identify the pathways that were induced or repressed during aging. To be noted, GSEA was performed on pseudobulk profiles aggregated across all cell clusters. Besides, some functional analyses were also performed using the OmicStudio tools, which are available at https://www.omicstudio.cn/tool.

### 5.7 STEM analysis

The STEM analysis was performed on the DEGs between pseudobulk profiles aggregated across all cell clusters during aging from CBMA or CBMD. The parameters of STEM were set as follows: *p* value ≤ 0.05 in Bonferroni's test and fold change ≥ 1.5 between any pair of different age groups from CBMA or CBMD. The maximal correlation between profiles was 0.7 for the functions “Modeling Profiles” and “Clustering Profiles.” Data acquired from the CBMA and CBMD groups were analyzed separately using STEM. Finally, DEGs passing the filters of STEM were processed for gene enrichment analysis using the GO annotations.

### 5.8 Pseudotime trajectory analysis

The R package Monocle2 (Trapnell et al., 2014) was used to infer the developmental trajectory of the mesenchymal cell cluster. After pseudotime was determined, the pseudotime kinetics of DEGs were displayed using the plot_genes_in_pseudotime function in the R package. The pesudotime heatmap was generated using the plot_pseudotime_heatmap function in the R package.

### 5.9 RNA velocity analysis

The RNA velocity was estimated using the velocyto.R package (La Manno et al., 2018), which indicated the future cell state. First, annotation of spliced and unspliced reads was conducted using velocyto.py command‐line tools. Next, we performed downstream analysis using the velocyto.R pipeline. The RNA velocity of each cell was estimated using the gene.relative.velocity.estimates function with default parameters. Finally, velocity fields and pseudotime values were projected onto the existing UMAP plot.

### 5.10 Cell–cell communication analysis

We utilized CellPhoneDB (Efremova et al., 2020) and CellChat (Jin et al., 2021) to analyze intercellular communication networks, which enabled the visualization and quantification of cellular interactions according to the expression level of ligand–receptor pairings. As for CellPhoneDB, the database is available at https://www.cellphonedb.org/. And the software package is available at https://github.com/Teichlab/cellphonedb. As for CellChat, the software package is available at https://github.com/sqjin/CellChat.

### 5.11 Statistical analysis

All data were presented as mean ± SD. Significance was assessed using two‐tailed Student's *t* test for comparison between two groups or one‐way analysis of variance (ANOVA) for comparison between more than two groups. A *p* value less than 0.05 was considered statistically significant.

## AUTHOR CONTRIBUTIONS

B. L., Y.L., and Z.Z. designed the study. B.L., J.L., and BZ.L. performed the experiments and analyzed the data. B.L. and J.L. generated the figures. B.L. drafted the manuscript. L.L., B.L., T.O., Y.L., and Z.Z. critically reviewed and edited the manuscript. All authors approved the final version of the manuscript.

## CONFLICT OF INTEREST STATEMENT

The authors declare no conflict of interest.

## Supporting information


FigureS1

FigureS2

FigureS3

FigureS4

FigureS5

FigureS6

FigureS7

FigureS8

FigureS9

FigureS10

## Data Availability

The data that support the findings of this study are openly available in NCBI Gene Expression Omnibus at https://www.ncbi.nlm.nih.gov/geo/, reference number [GSE235176] (Li et al., 2023).
