# Supplementary figures and images for: A single‐cell transcriptomic atlas characterizes age‐related changes of murine cranial stem cell niches
Source: Aging Cell. 2023 Sep 8;22(11):e13980. doi: 10.1111/acel.13980 (PMC10652347; doi:10.1111/acel.13980)

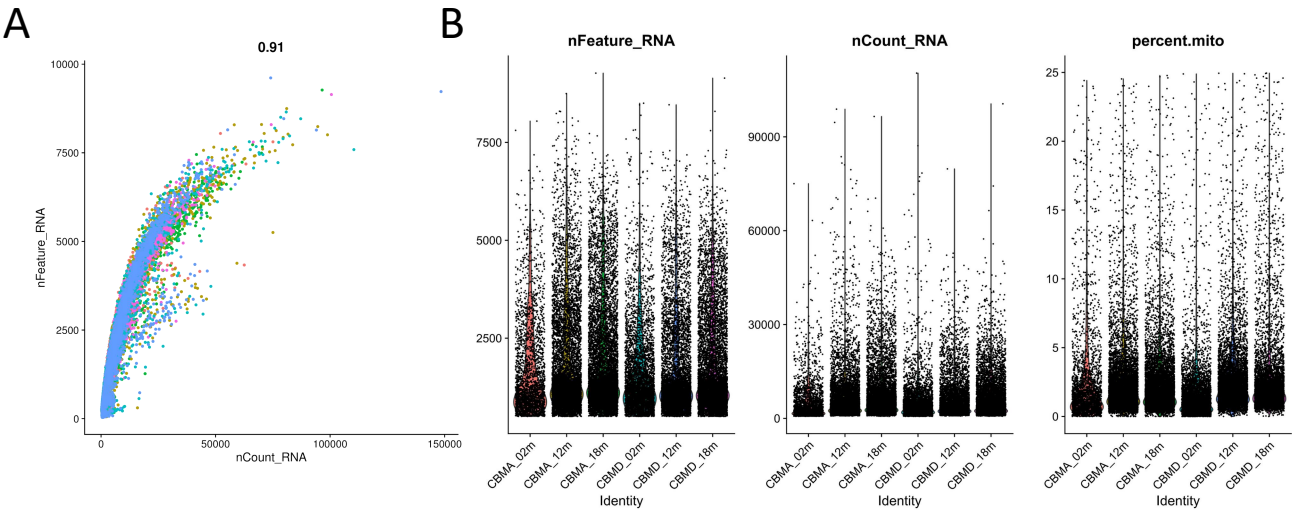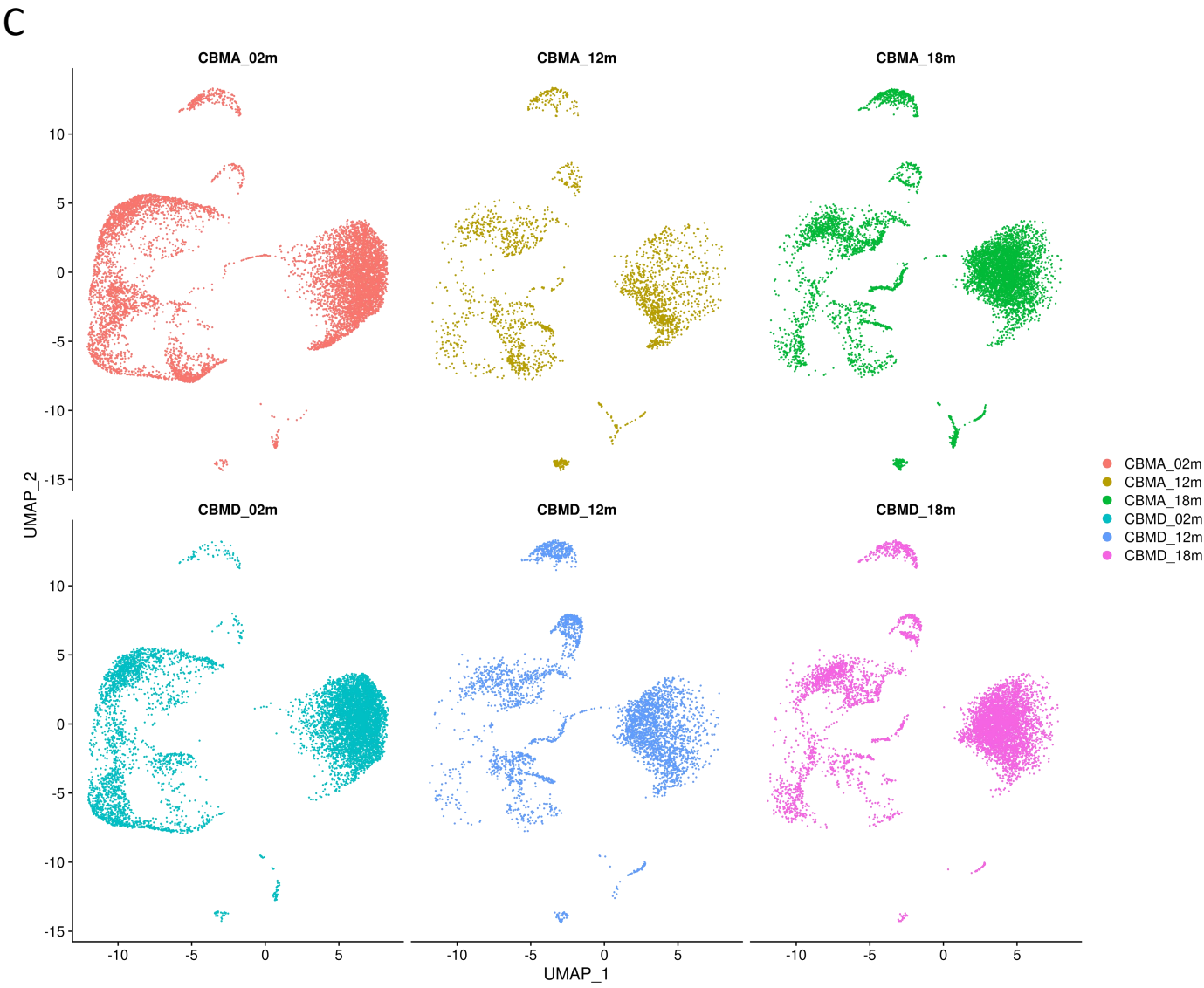

Supplement: Supplementary file 1 — FigureS1 [file ACEL-22-e13980-s005.pdf]

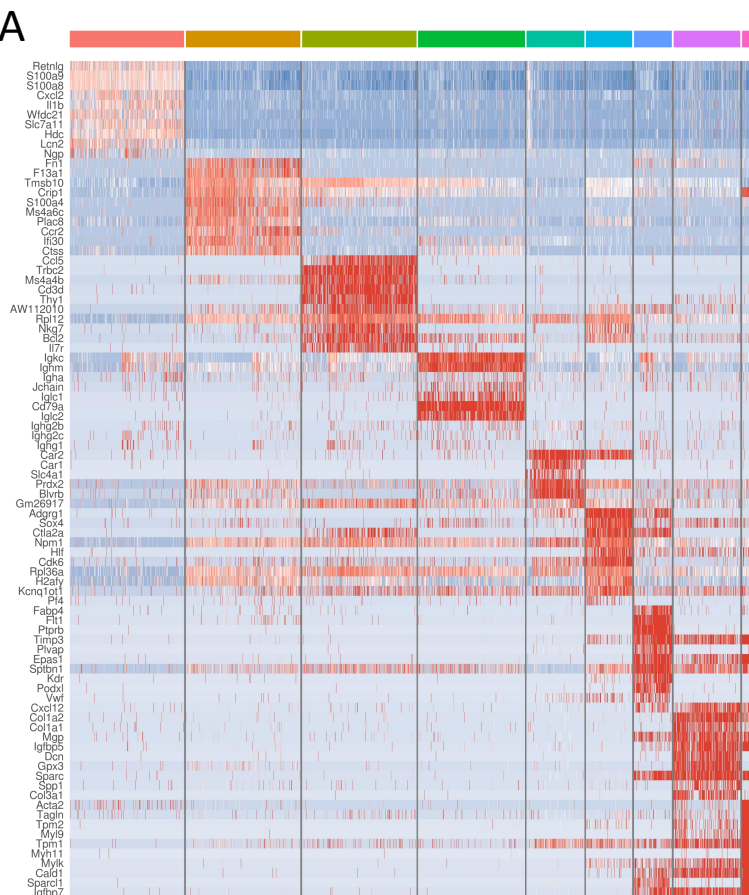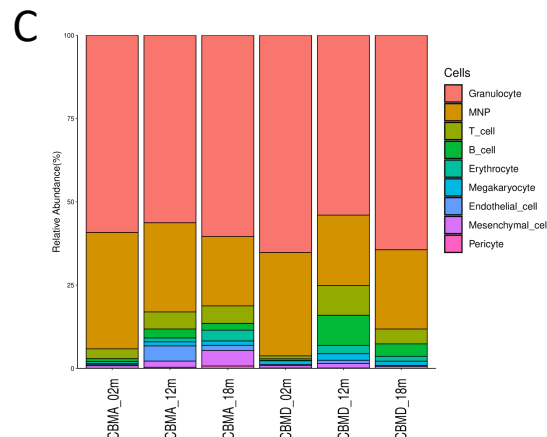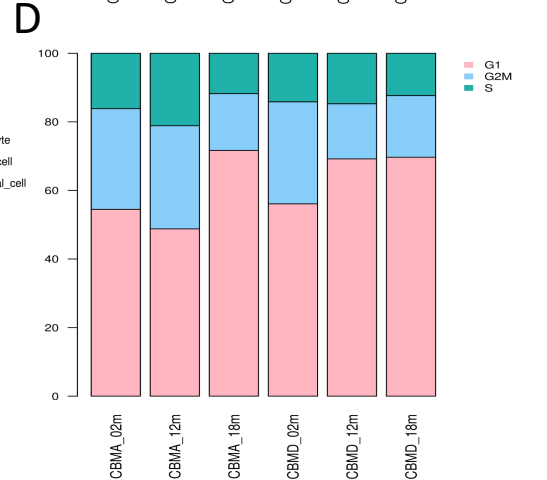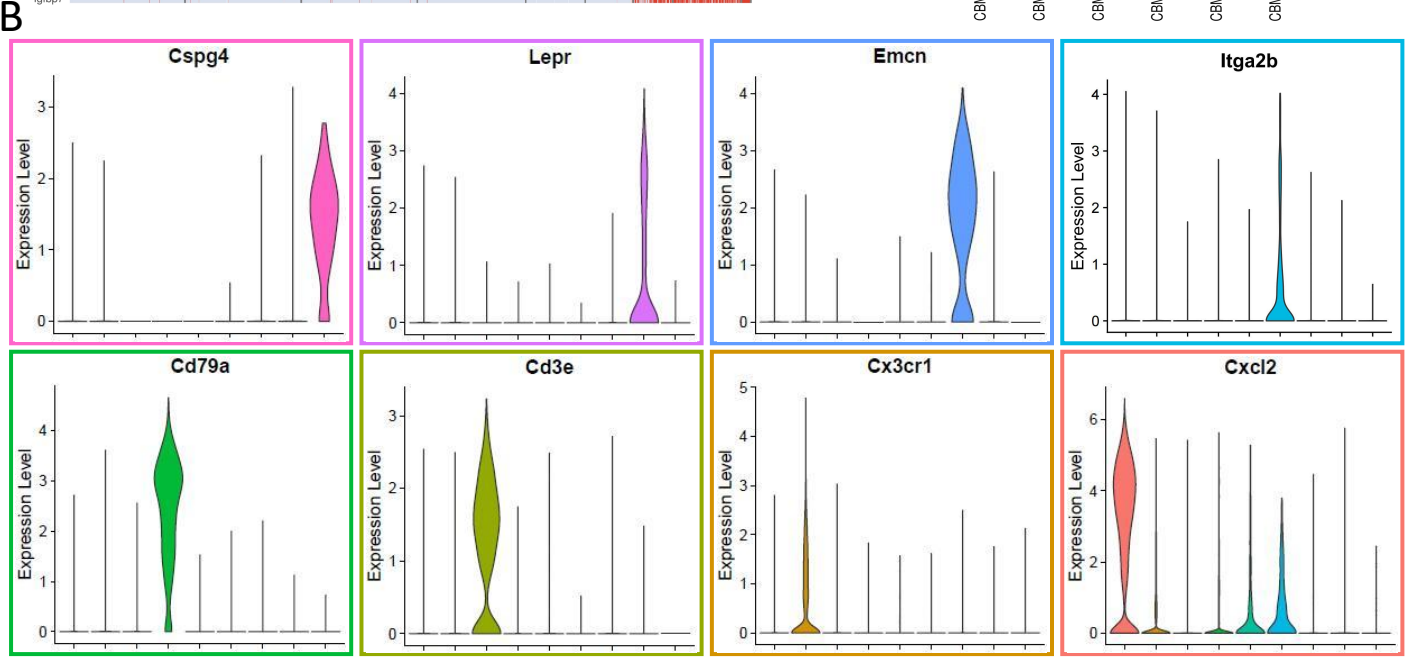

Supplement: Supplementary file 2 — FigureS2 [file ACEL-22-e13980-s002.pdf]

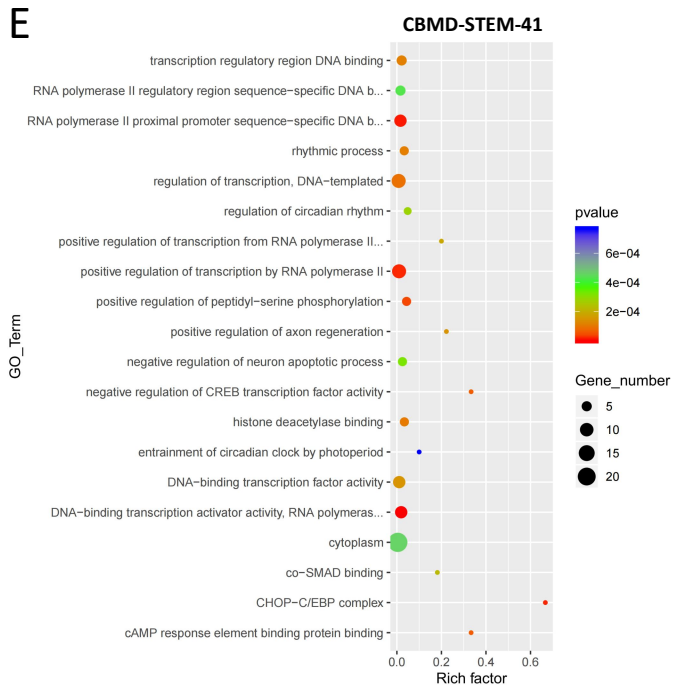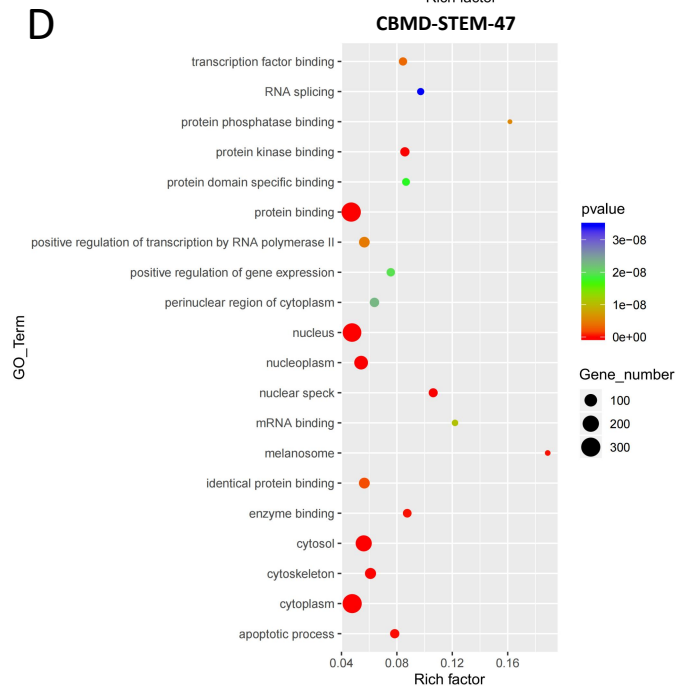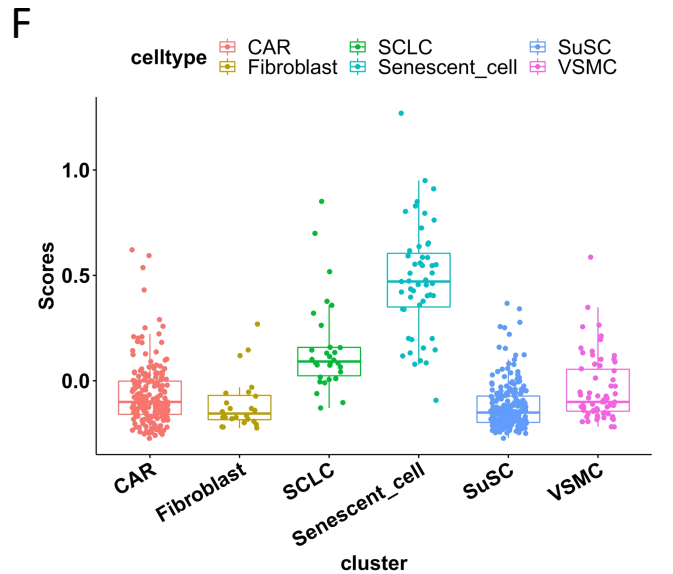

Supplement: Supplementary file 3 — FigureS3 [file ACEL-22-e13980-s006.pdf]

A

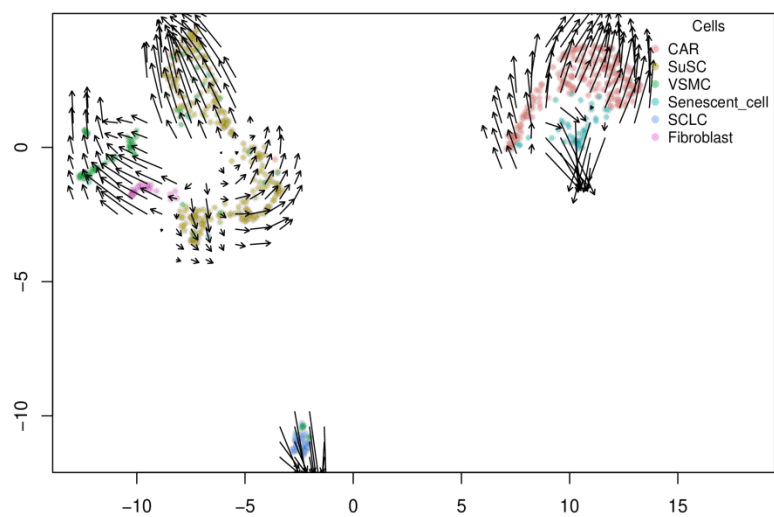

B

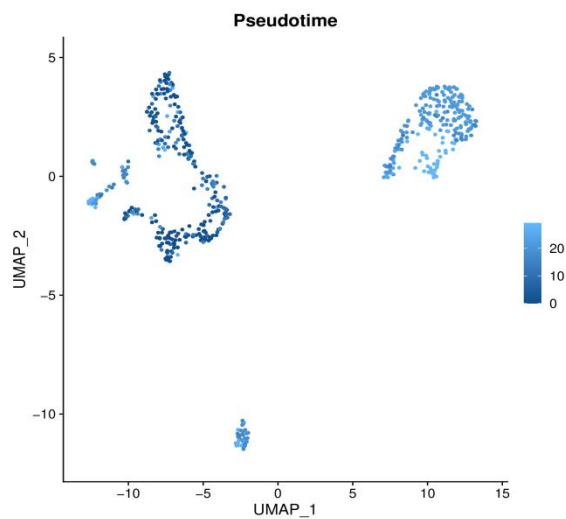

C

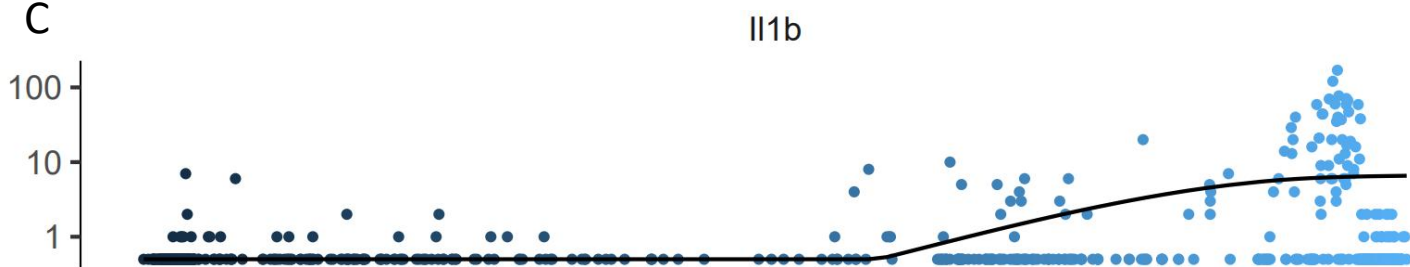

D

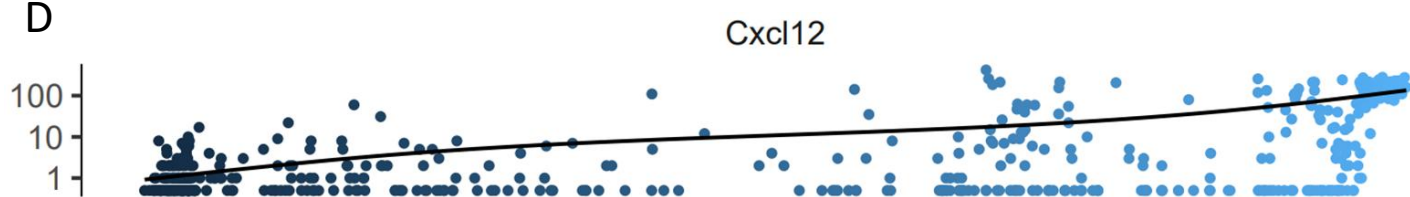

E

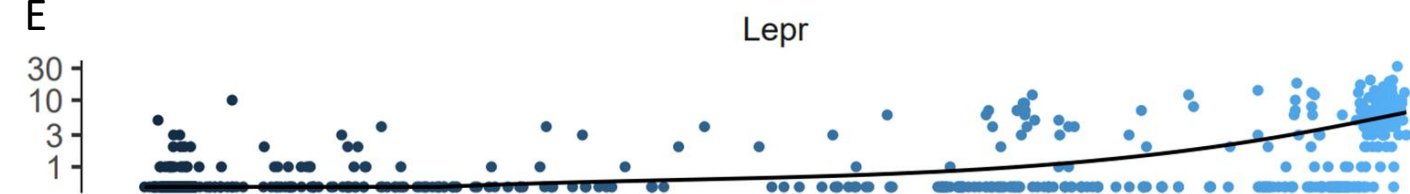

F

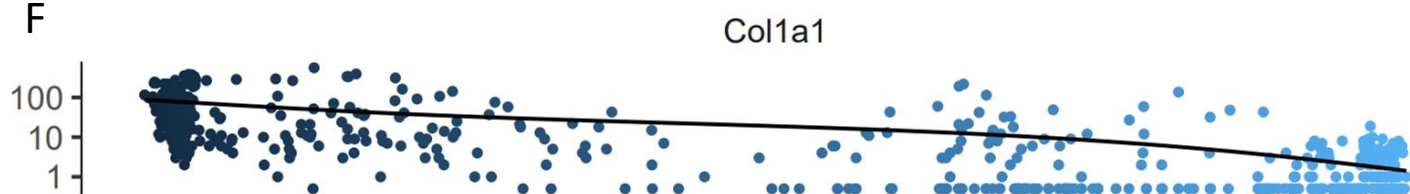

Supplement: Supplementary file 4 — FigureS4 [file ACEL-22-e13980-s008.pdf]

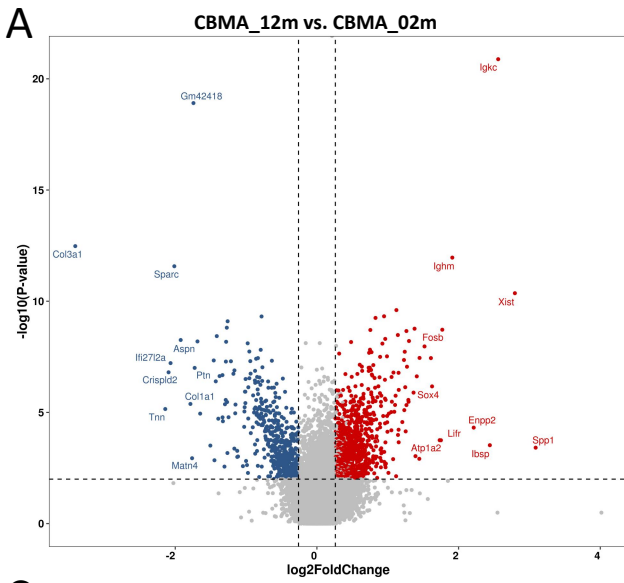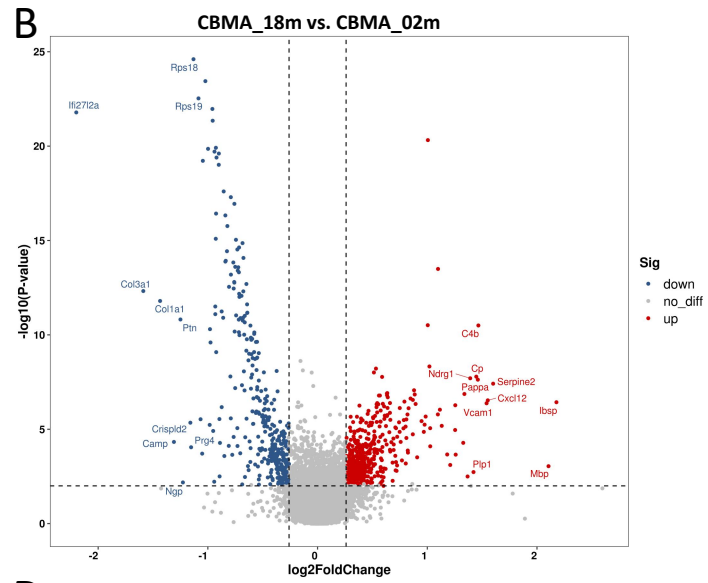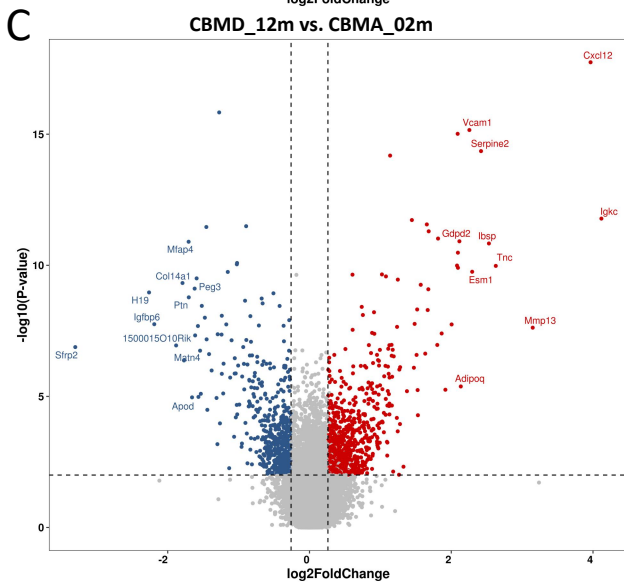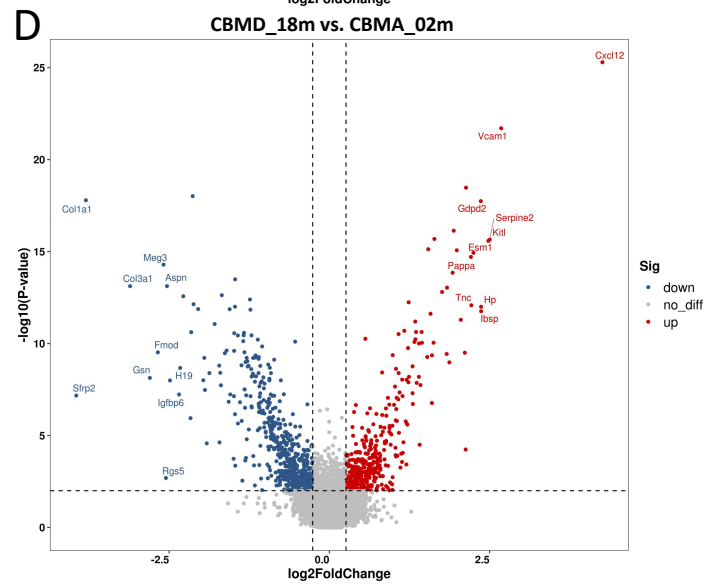

Supplement: Supplementary file 5 — FigureS5 [file ACEL-22-e13980-s004.pdf]

A

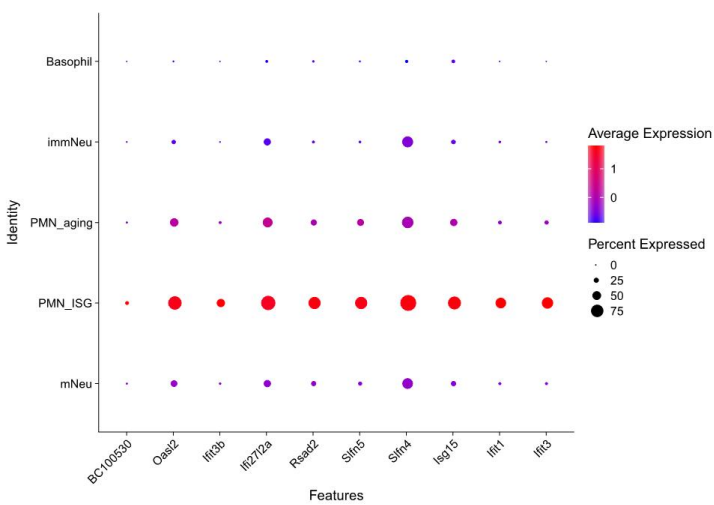

B

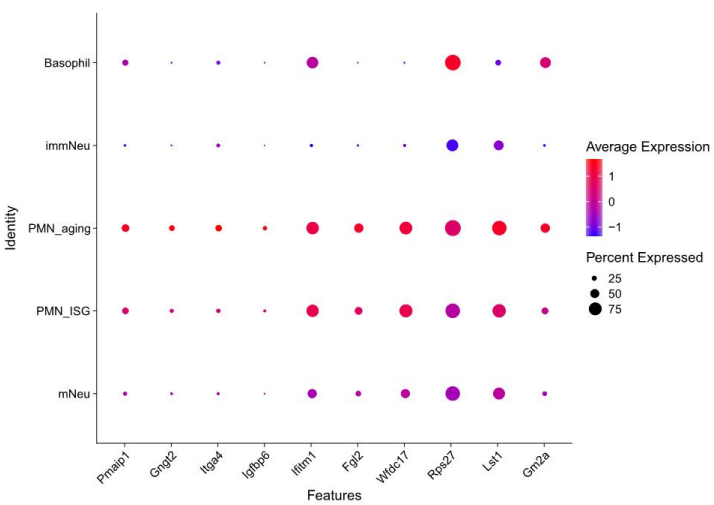

C

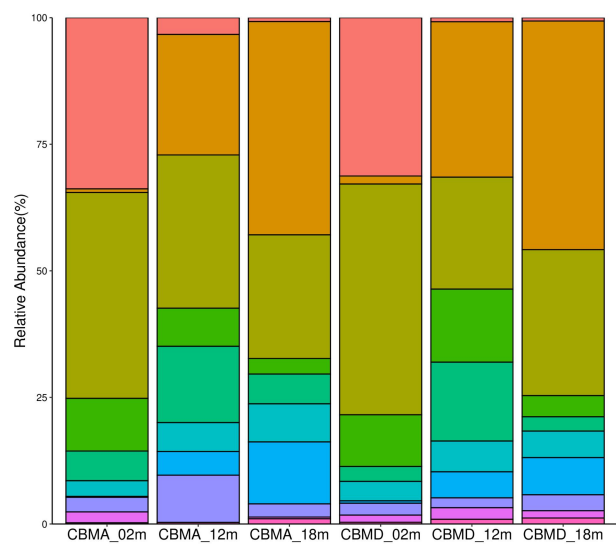

D

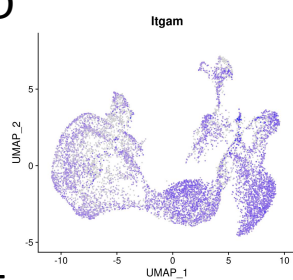

E

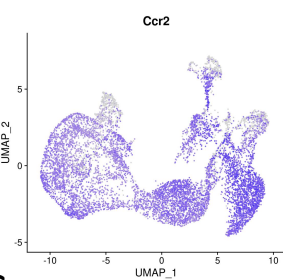

F

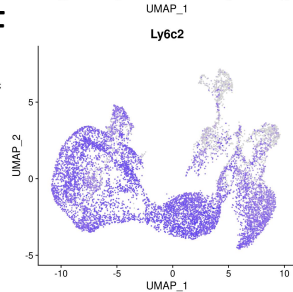

G

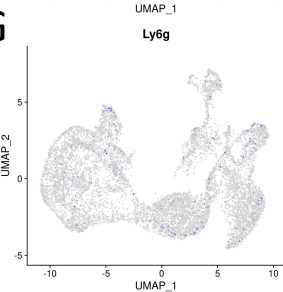

H

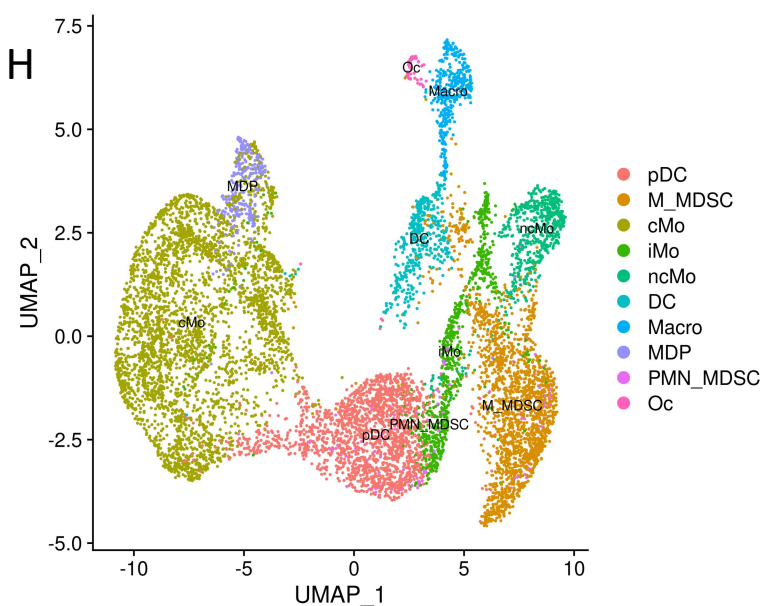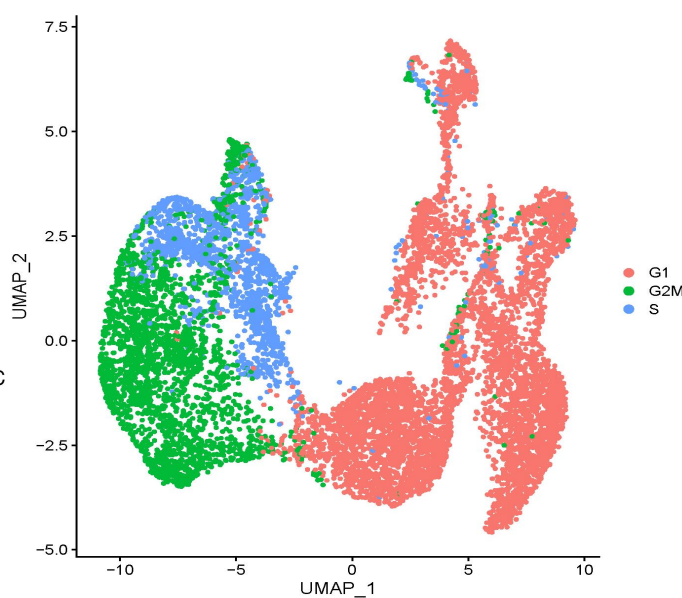

Supplement: Supplementary file 6 — FigureS6 [file ACEL-22-e13980-s003.pdf]

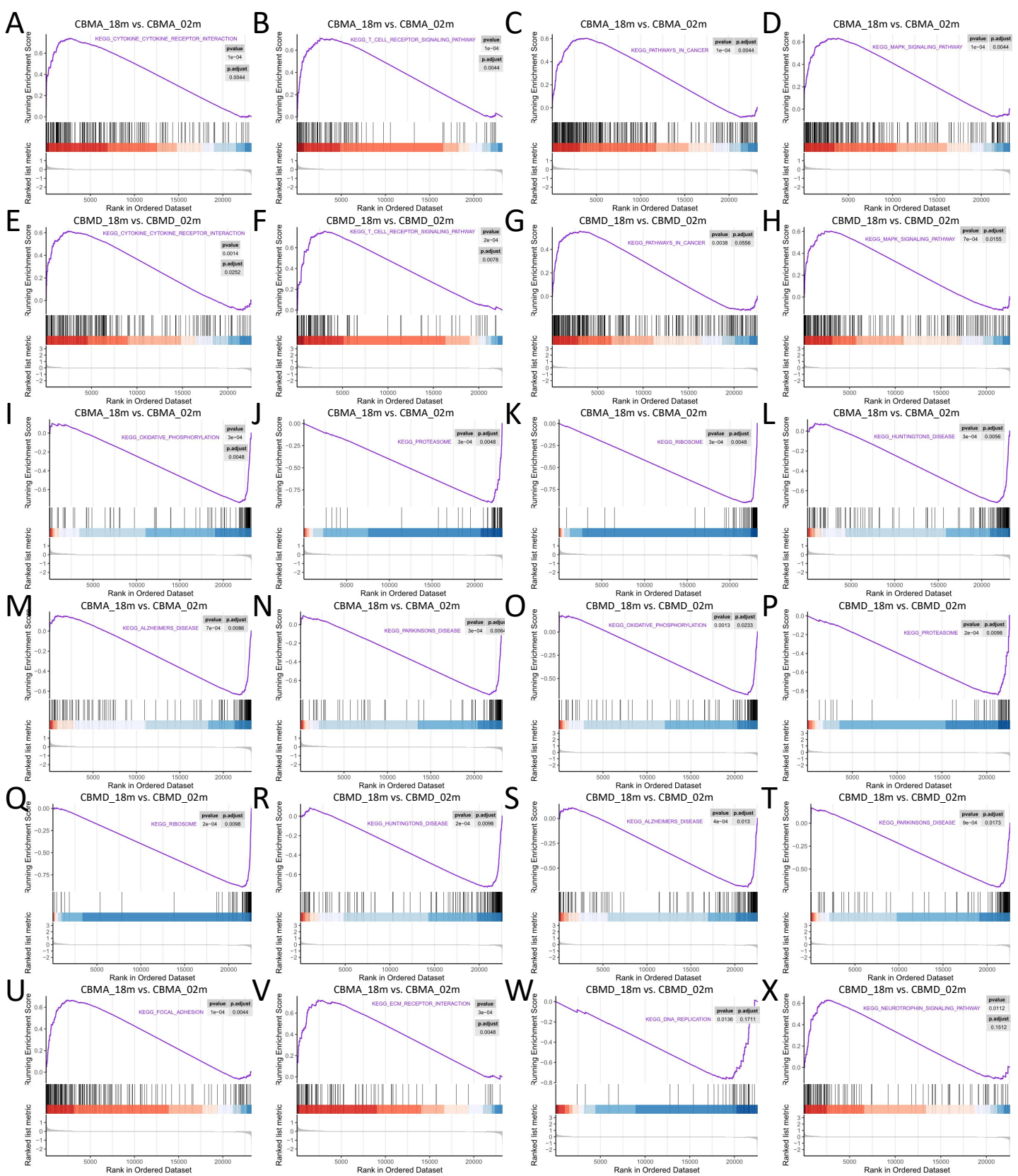

Supplement: Supplementary file 7 — FigureS7 [file ACEL-22-e13980-s009.pdf]

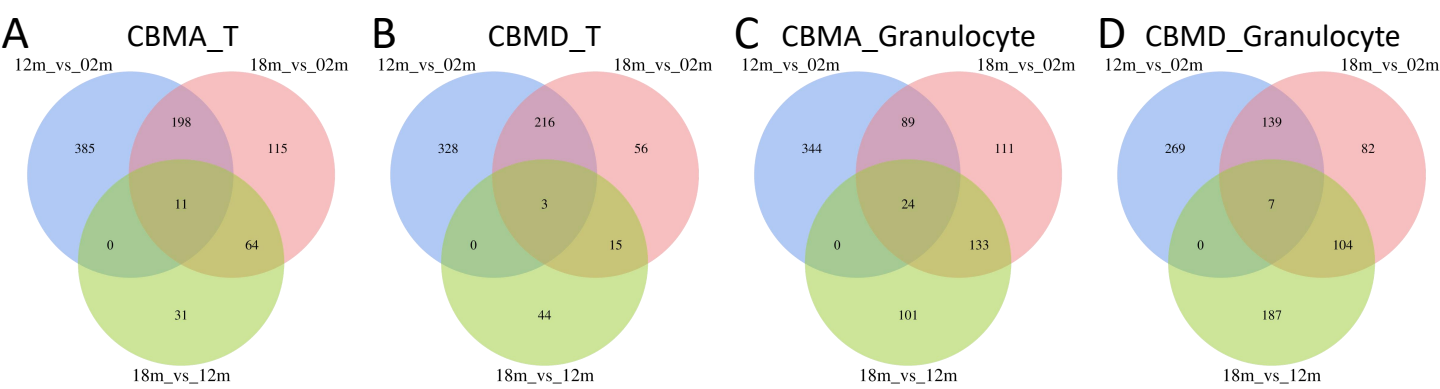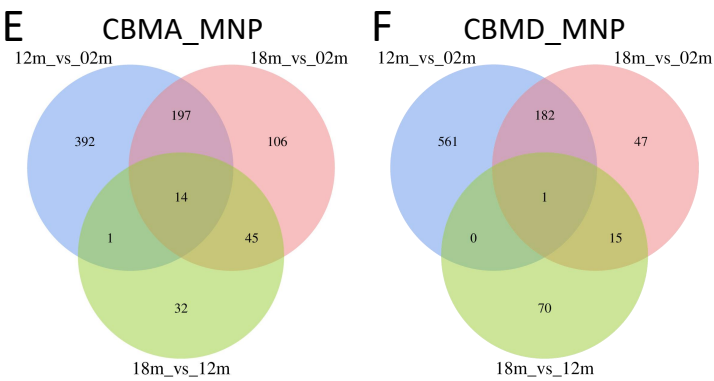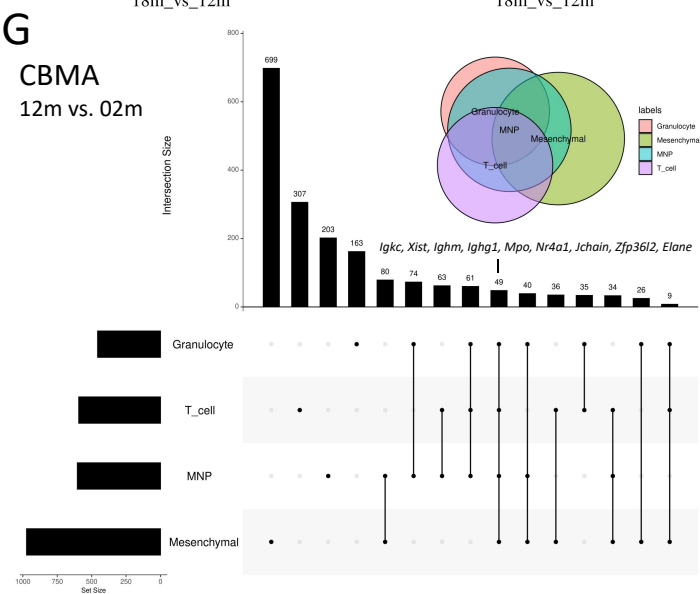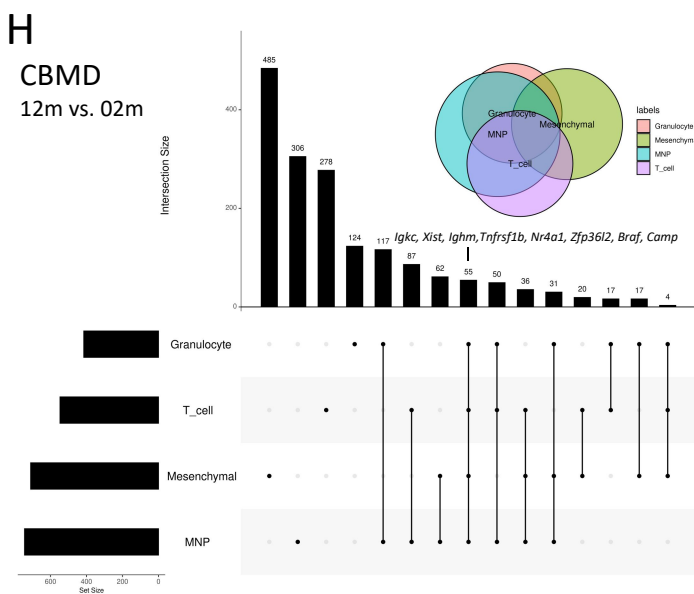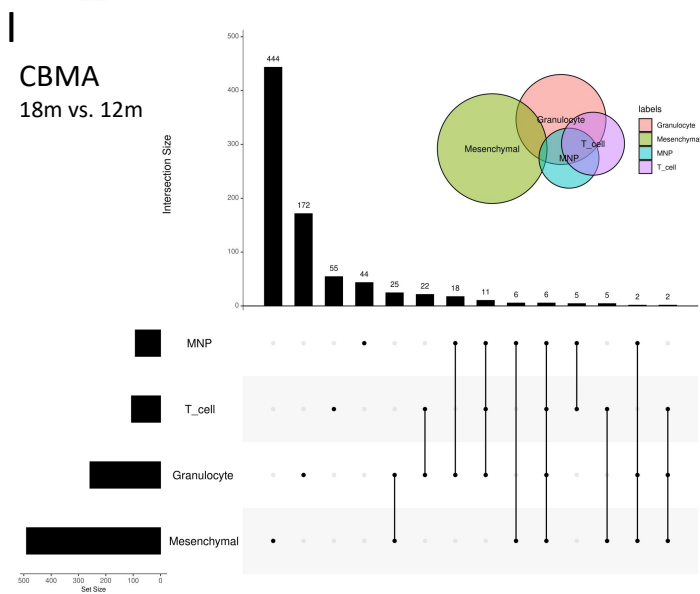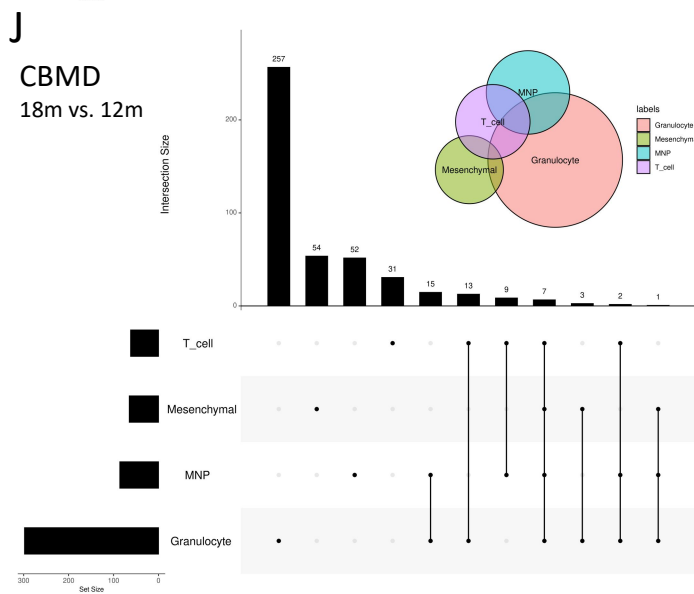

Supplement: Supplementary file 8 — FigureS8 [file ACEL-22-e13980-s007.pdf]

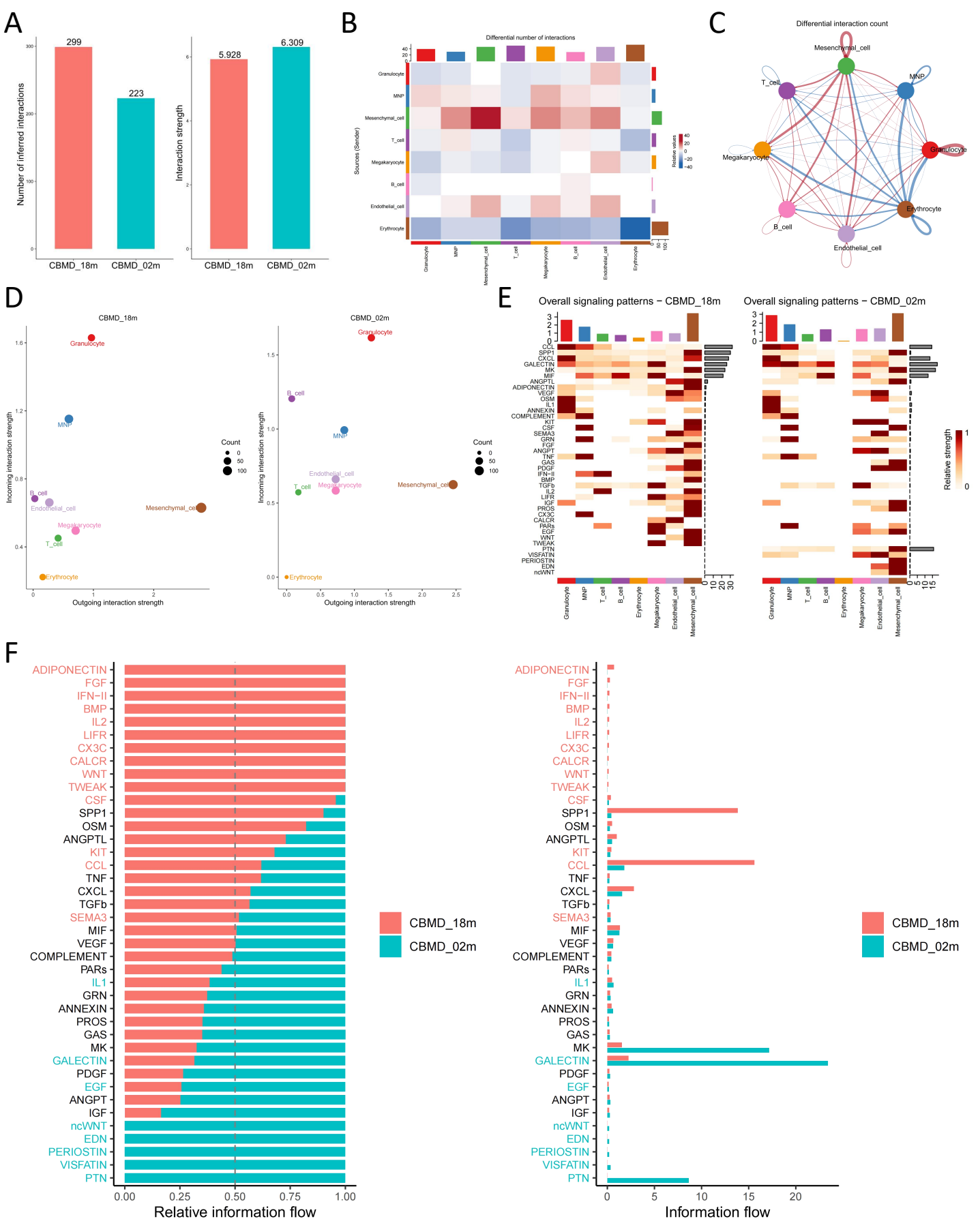

Supplement: Supplementary file 10 — FigureS10 [file ACEL-22-e13980-s010.pdf]
