# Supplementary material for: A single‐cell transcriptomic atlas characterizes age‐related changes of murine cranial stem cell niches
Source: Aging Cell. 2023 Sep 8;22(11):e13980. doi: 10.1111/acel.13980 (PMC10652347; doi:10.1111/acel.13980)
Supplement: Supplementary file 9 — FigureS9 [file ACEL-22-e13980-s001.pdf]

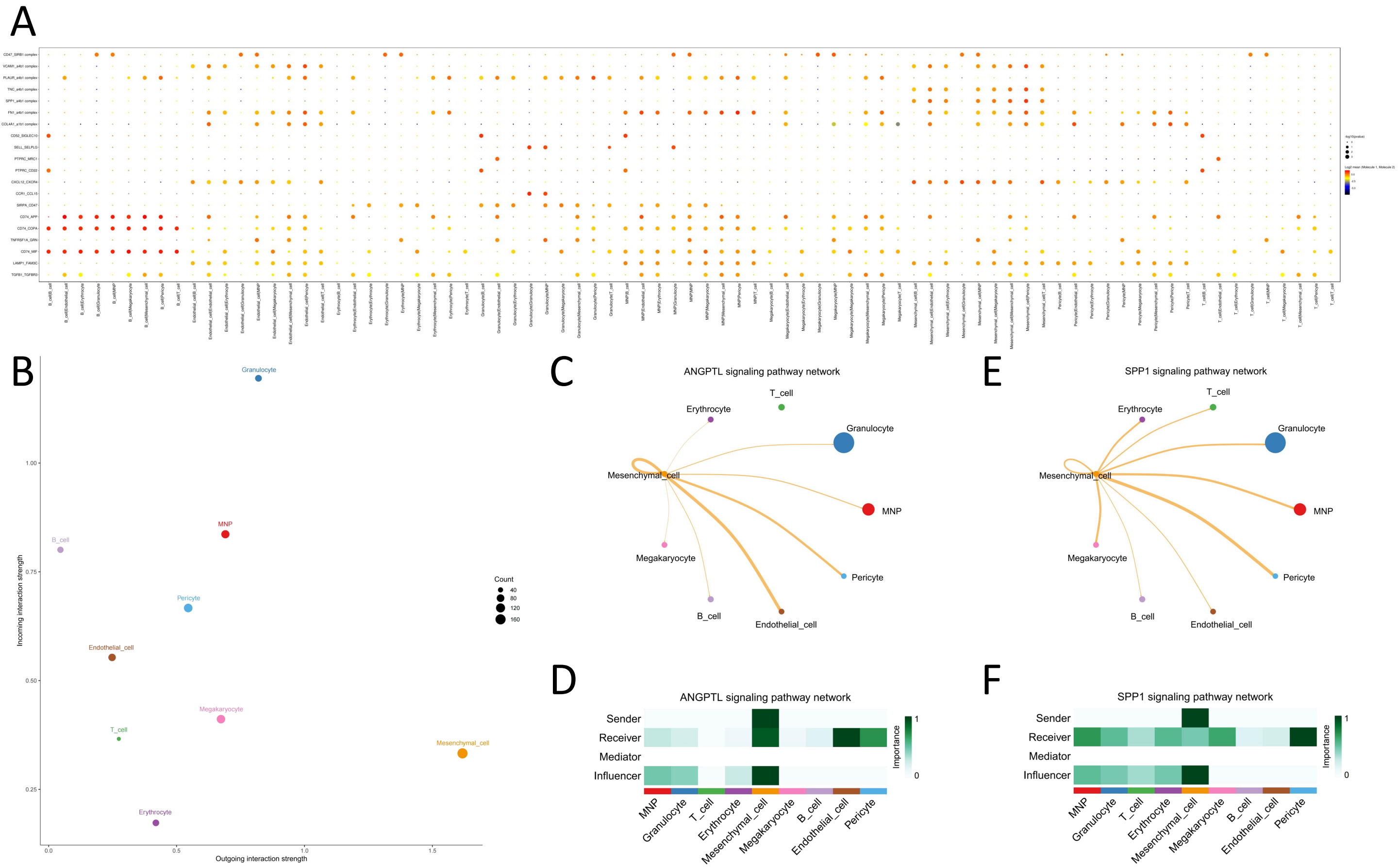

A
B
Granulocyte
MNP
B_cell
Pericyte
Endothelial_cell
Megakaryocyte
T_cell
Mesenchymal_cell
Erythrocyte
Incoming interaction strength
Outgoing interaction strength
Count
40
80
120
160
1.00
0.75
0.50
0.25
0.0
0.5
1.0
1.5
C
ANGPTL signaling pathway network
T_cell
Erythrocyte
Granulocyte
Mesenchymal_cell
MNP
Megakaryocyte
Pericyte
B_cell
Endothelial_cell
D
ANGPTL signaling pathway network
Sender
Receiver
Mediator
Influencer
Importance
1
0
MNP
Granulocyte
T_cell
Erythrocyte
Mesenchymal_cell
Megakaryocyte
B_cell
Endothelial_cell
Pericyte
E
SPP1 signaling pathway network
T_cell
Erythrocyte
Granulocyte
Mesenchymal_cell
MNP
Megakaryocyte
Pericyte
B_cell
Endothelial_cell
F
SPP1 signaling pathway network
Sender
Receiver
Mediator
Influencer
Importance
1
0
MNP
Granulocyte
T_cell
Erythrocyte
Mesenchymal_cell
Megakaryocyte
B_cell
Endothelial_cell
Pericyte
